# Supplementary material for: circ_PTN contributes to -cisplatin resistance in glioblastoma via PI3K/AKT signaling through the miR-542-3p/PIK3R3 pathway
Source: Mol Ther Nucleic Acids. 2021 Sep 7;26:1255–69. doi: 10.1016/j.omtn.2021.08.034 (PMC8607136; doi:10.1016/j.omtn.2021.08.034)
Supplement: Document S1. Figures S1–S5 and Tables S1–S2 [file mmc1.pdf]

OMTN, Volume *26*

Supplemental information

circ_PTN contributes to -cisplatin resistance in glioblastoma via PI3K/AKT signaling through the miR-542-3p/PIK3R3 pathway

Hongcheng Luo, Tingzhuang Yi, Deyou Huang, Xiaoping Chen, Xu Li, Qianquan Wan, Haineng Huang, Huadong Huang, Hongyu Wei, Ye Song, Tianshi Que, Rentong Hu, Huatuo Huang, Kunxiang Luo, Chuanyu Li, Chengjian Qin, Chuanhua Zheng, Chuanliu Lan, Wencheng Chen, Dan Zhou, and Qisheng Luo

Supplementary Table 1 Information on the antibodies was presented.

Supplementary Table 1. Information on the antibodies

Antibody	Catalog number	Dilution	Source
Bcl-2	#4223	1/1000	CST, Boston, MA, USA
Caspase-3	#9662	1/1000	CST, Boston, MA, USA
Cleaved Caspase-3	#9661	1/1000	CST, Boston, MA, USA
Caspase-9	#9502	1/1000	CST, Boston, MA, USA
Cleaved Caspase-9	#9505	1/1000	CST, Boston, MA, USA
β-actin	ab8226	1/1000	Abcam, Cambridge, MA, USA
PIK3R3	27035-1-AP	1/500	Proteintech, Chicago, IL, USA
GAPDH	#5174	1/1000	CST, Boston, MA, USA
PI3K-alpha	#4255	1/1000	CST, Boston, MA, USA
p-AKT	ab38449	1/500	Abcam, Cambridge, MA, USA
AKT	ab8805	1/500	Abcam, Cambridge, MA, USA
secondary antibody	ab205718	1/10000	Abcam, Cambridge, MA, USA

Supplementary Table 2 Database accession numbers of new gene/protein sequences were provided.

Supplementary Table 2. Accession Number (Sequences of Genes/Proteins)

**Gene/Protein Name**	**Accession Number of Sequence**
circ_COL1A2	NM_000089
circ_VCAN	NM_004385
circ_PTN	NM_002825
circ_SMO	NM_005631
circ_PLOD2	NM_182943
circ_GLIS3	NM_001042413
circ_EPHB4	NM_004444
circ_CLIP2	NM_003388
Bcl-2	Q92934
Caspase-3	P42574
Cleaved Caspase-3	P42574

Caspase-9	P55211
Cleaved Caspase-3	P55211
β-actin	P60709
CDR1as	NM_004065
cANRIL	NR_003529.3
hsa-miR-223-3p	MIMAT0004570
hsa-miR-432-5p	MIMAT0002814
hsa-miR-620	MIMAT0003289
hsa-miR-1270	MIMAT0005924
hsa-miR-542-3p	MIMAT0003340
AKT1	NM_005163.2
AKT2	NM_001626.6
TSC1	NM_000368.5
IGF1R	NM_000875.5
PIK3R1	NM_181523.3
PIK3R3	NM_003629.4

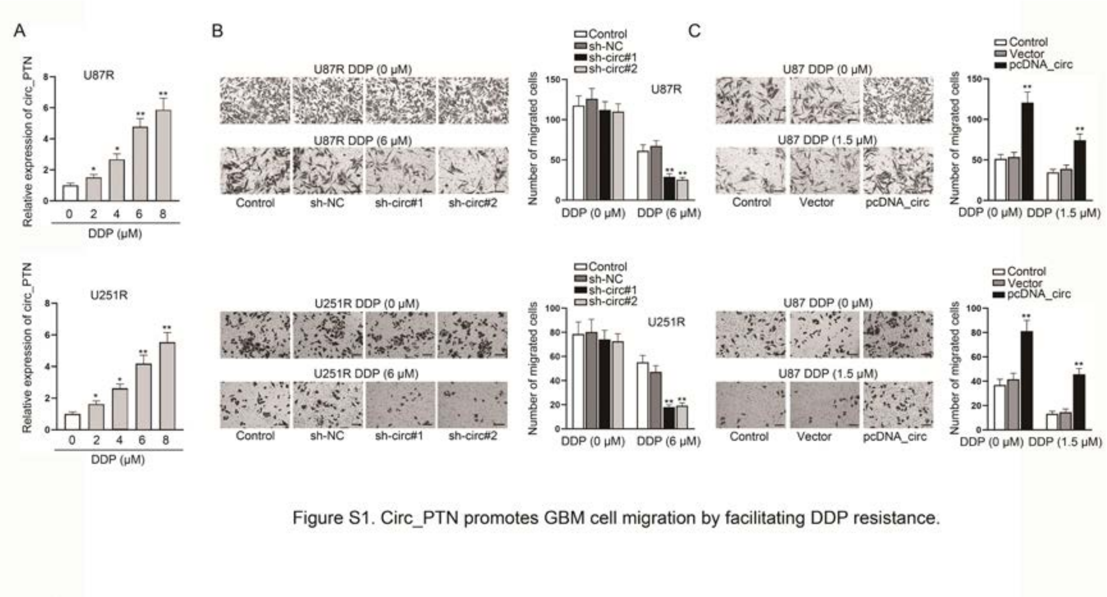

Figure S1. Circ_PTN promotes GBM cell migration by facilitating DDP resistance.

(A). RT-qPCR assay was adopted to detect the RNA level of circ_PTN in DDP-resistant GBM cells treated by different doses of DDP. **(B-C).** Transwell assay

was conducted to test the migration of U87R and U251R cells treated by different doses of DDP upon circ_PTN silencing. *P<0.05, **P<0.01.

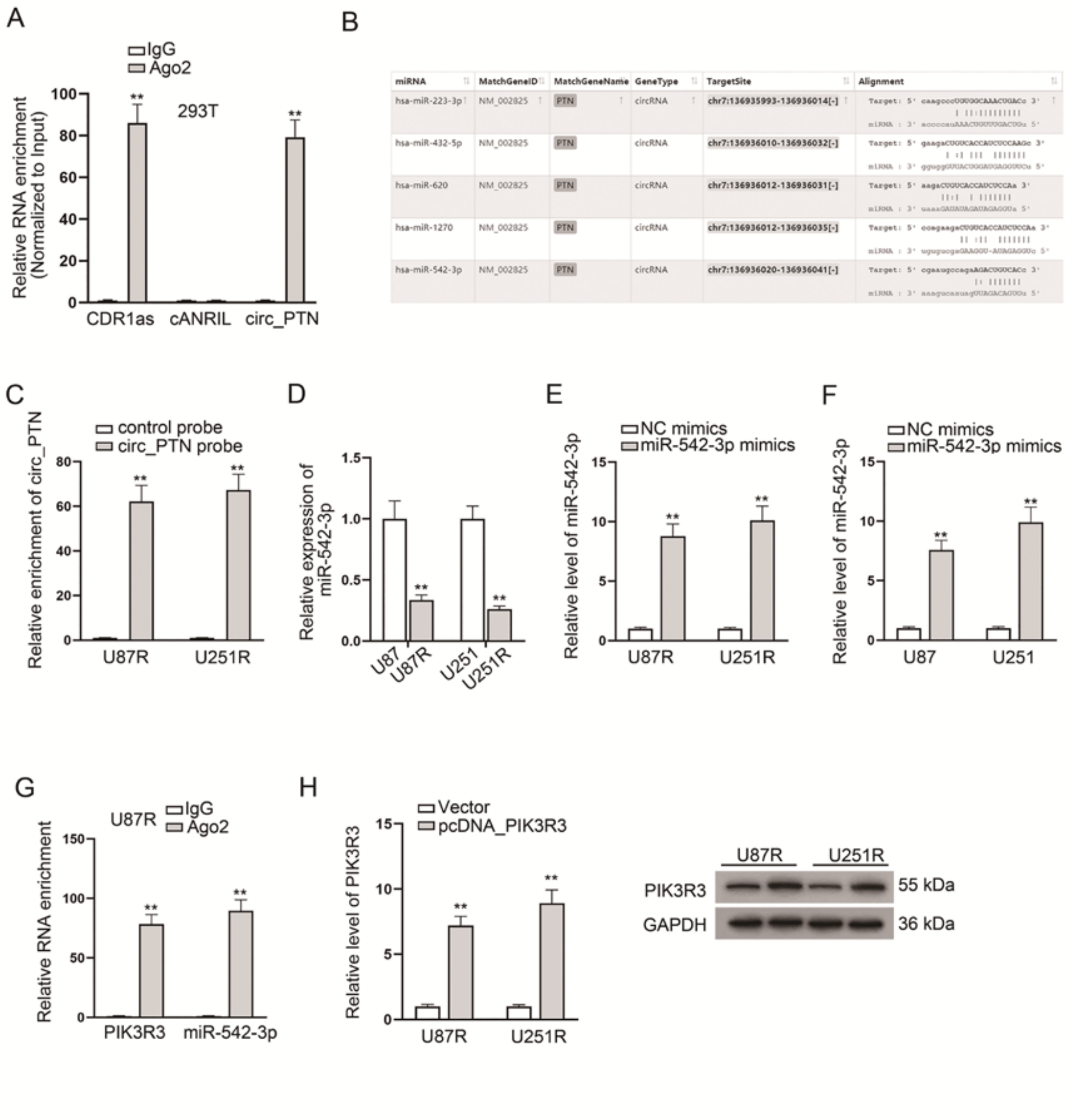

Figure S2. Circ_PTN sponges miR-542-3p to augment PIK3R3 in GBM cells.

Figure S2. Circ_PTN sponges miR-542-3p to augment PIK3R3 in GBM cells.

(A). RIP assay was used to detect the enrichment of CDR1as, cANRIL and circ_PTN in Ago2 groups. **(B).** StarBase was used to predict the miRNAs which could interact with circ_PTN. **(C).** RNA pull down assay was adopted to detect the enrichment of circ_PTN pulled down by circ_PTN probes in DDP-resistant GBM cells. **(D).**

RT-qPCR assay was utilized to analyze the RNA level of miR-542-3p in DDP-resistant GBM cells. **(E-F).** RT-qPCR assay was adopted to examine the RNA level of miR-542-3p in DDP-resistant GBM cells after the transfection with miR-542-3p mimics. **(G).** RIP assay was conducted to analyze the enrichment of PIK3R3 and miR-542-3p in Ago2 groups. **(H).** RT-qPCR and western blot assays were used to examine the mRNA and protein levels of PIK3R3 in DDP-resistant GBM cells. **$P<0.01$.

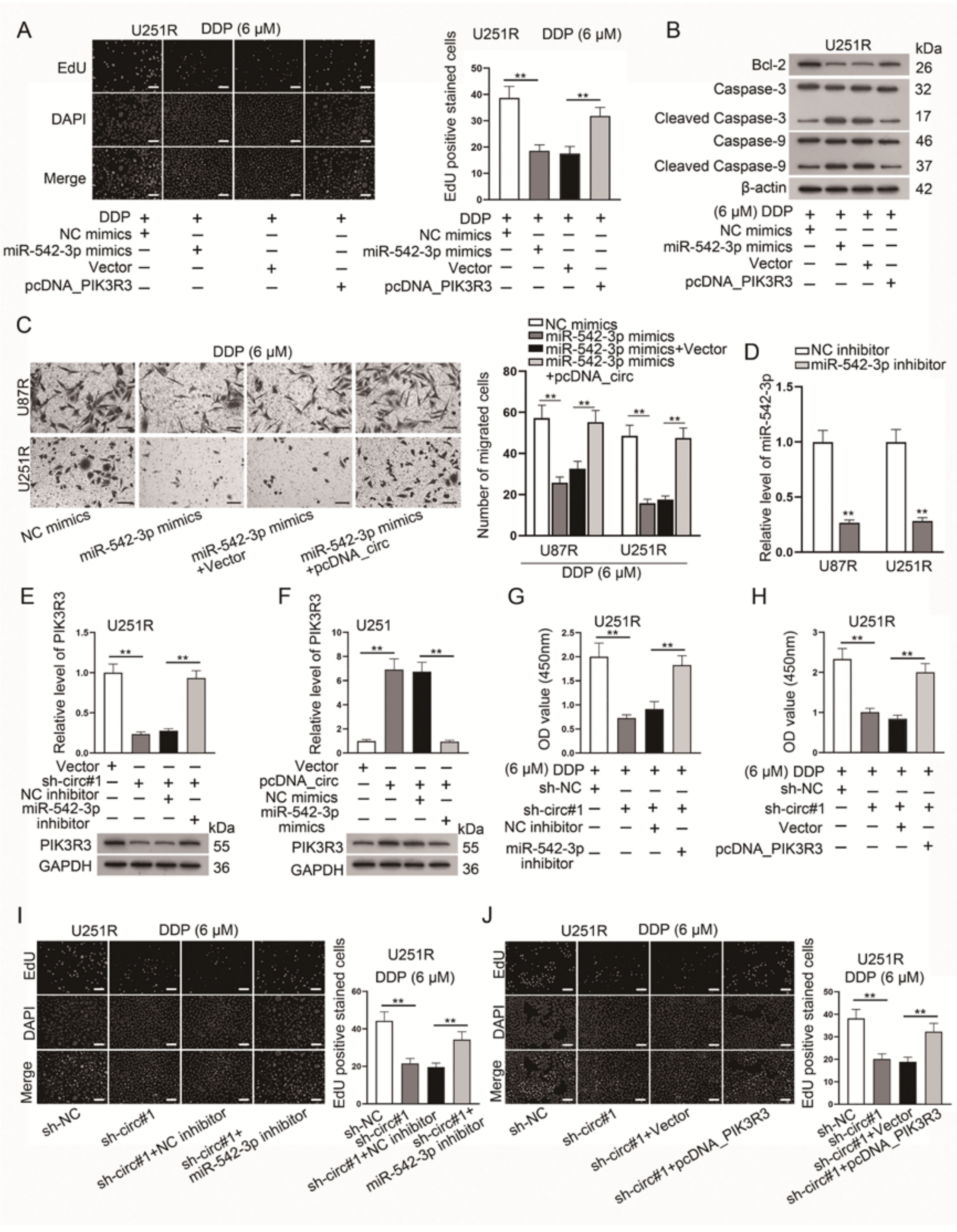

Figure S3. Circ_PTN regulates the DDP resistance via miR-542-3p/PIK3R3 signaling.

Figure S3. Circ_PTN regulates the DDP resistance via miR-542-3p/PIK3R3 signaling.

(A-B). EdU and western blot assays were used to detect proliferation of U251R cells

in different transfection groups and after DDP (6 μM) treatment. **(C).** Transwell assay was utilized to detect the migration of DDP-resistant GBM cells in different transfection groups after the treatment of 6 μM DDP. **(D).** RT-qPCR assay was used to test the RNA level of miR-542-3p in DDP-resistant GBM cells in different transfection groups. **(E-F).** RT-qPCR and western blot assays were used to detect the mRNA and protein levels of PIK3R3 in U251R and U251 cells in different transfection groups. **(G-H).** CCK-8 assays were adopted to examine the viability of DDP-resistant U251R cells in different transfection groups after the treatment of 6 μM DDP. **(I-J).** EdU assays were utilized to detect the proliferation of U251R cells in different transfection groups after the treatment of 6 μM DDP. **$P<0.01$.

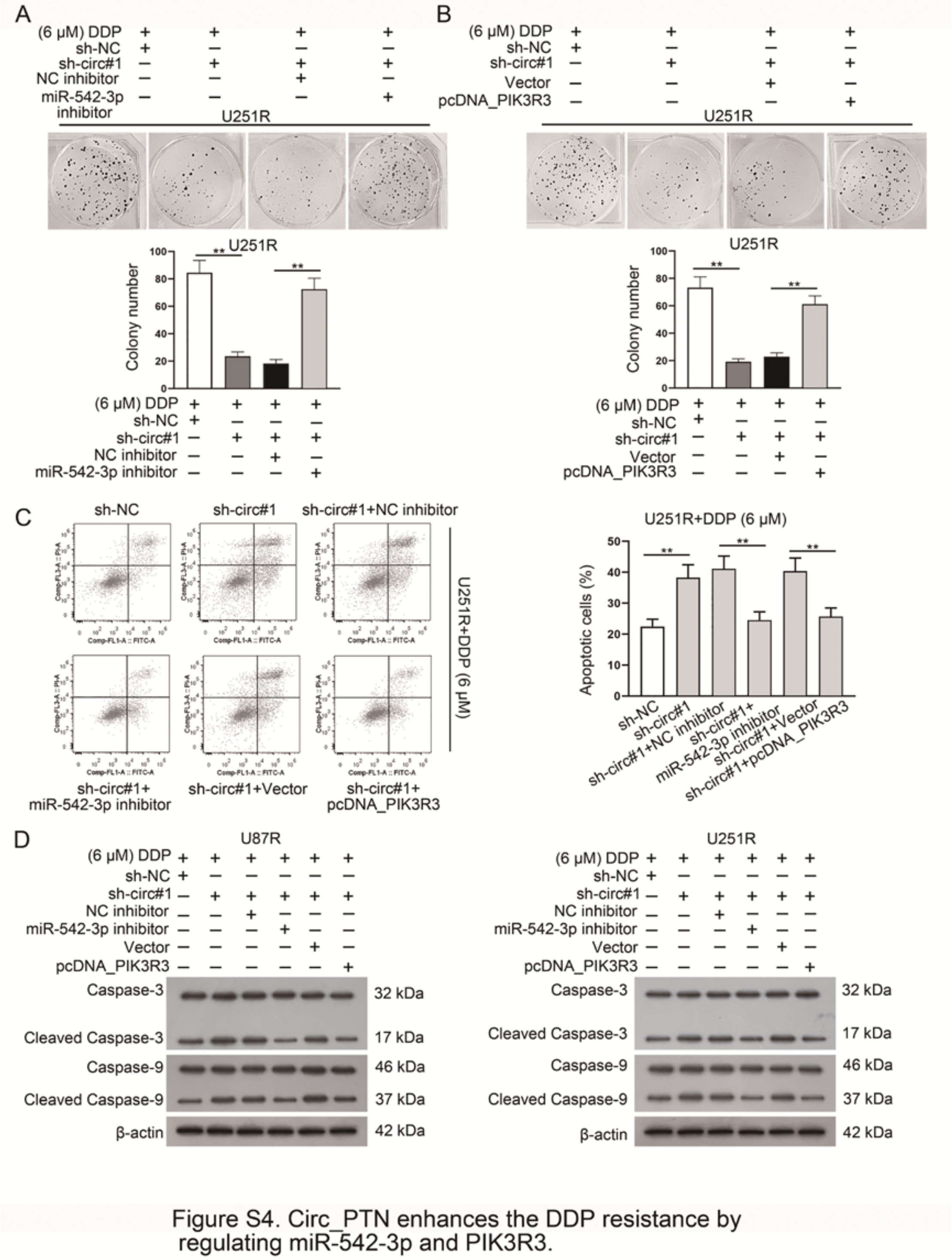

Figure S4. Circ_PTN enhances the DDP resistance by regulating miR-542-3p and PIK3R3.

(A-B). Colony formation assays were conducted to examine the proliferation of U251R cells in different transfection groups after the treatment of 6 μM DDP. **(C-D).**

Flow cytometry and western blot assays were carried out to examine the apoptosis of DDP-treated U251R cells under different transfection conditions. **P<0.01.

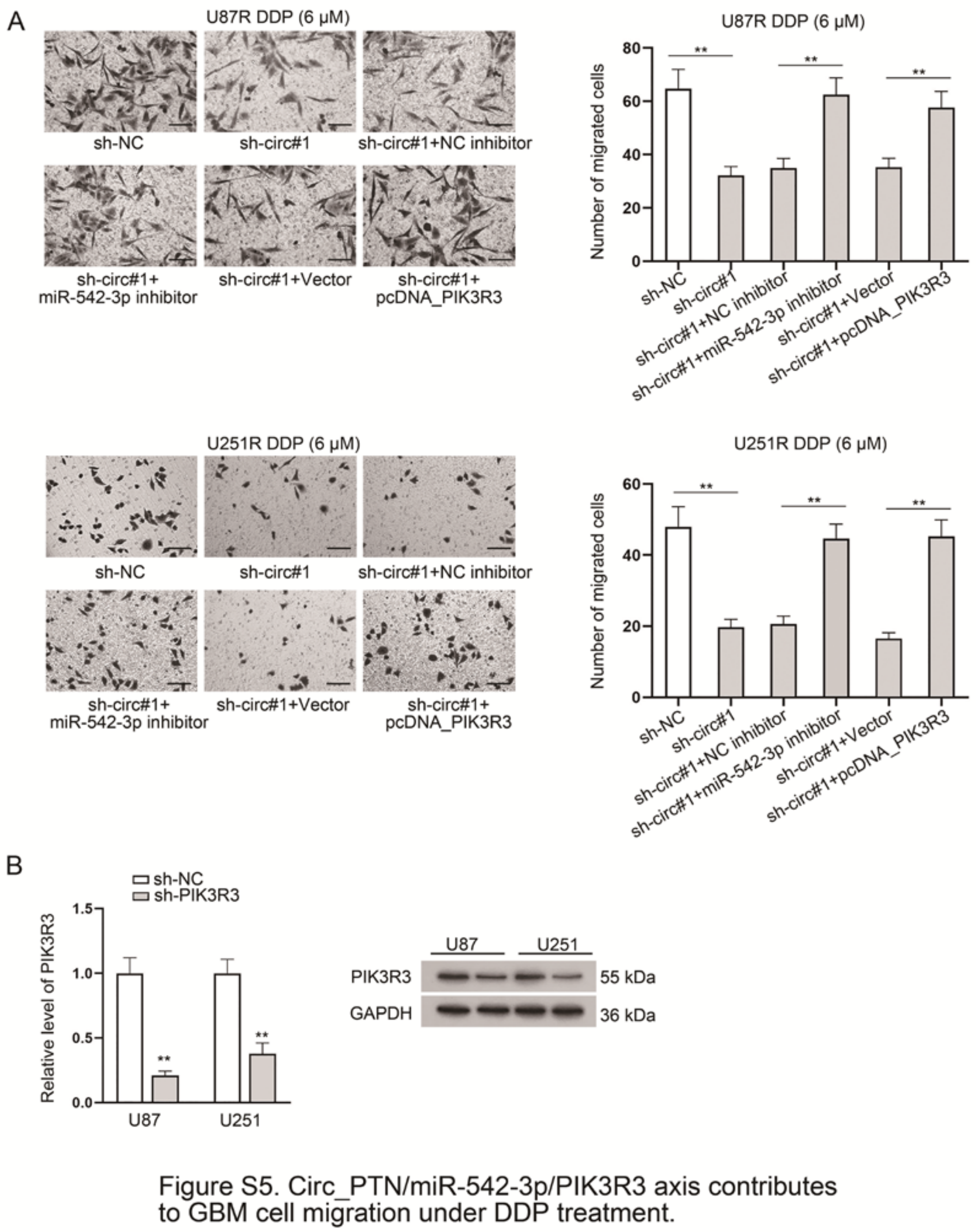

Figure S5. Circ_PTN/miR-542-3p/PIK3R3 axis contributes to GBM cell migration under DDP treatment.

(A). Transwell assays were used to analyze the migration of DDP-resistant GBM cells in different transfection groups after the treatment of 6 μM DDP. **(B).** RT-qPCR and

western blot assays were utilized to test the mRNA and protein levels of PIK3R3 in U87 and U251 cells transfected with sh-NC or sh-PIK3R3. **P<0.01.
