# Supplementary material for: circ_PTN contributes to -cisplatin resistance in glioblastoma via PI3K/AKT signaling through the miR-542-3p/PIK3R3 pathway
Source: Mol Ther Nucleic Acids. 2021 Sep 7;26:1255–69. doi: 10.1016/j.omtn.2021.08.034 (PMC8607136; doi:10.1016/j.omtn.2021.08.034)
Supplement: Document S2. Article plus supplemental information [file mmc2.pdf]

Molecular Therapy
Nucleic Acids
Original Article

circ_PTN contributes to -cisplatin resistance in glioblastoma via PI3K/AKT signaling through the miR-542-3p/PIK3R3 pathway

Hongcheng Luo,[1,11] Tingzhuang Yi,[2,11] Deyou Huang,[3,11] Xiaoping Chen,[4,11] Xu Li,[5] Qianquan Wan,[6] Haineng Huang,[7] Huadong Huang,[7] Hongyu Wei,[8] Ye Song,[9] Tianshi Que,[9] Rentong Hu,[1] Huatuo Huang,[1] Kunxiang Luo,[7] Chuanyu Li,[7] Chengjian Qin,[7] Chuanhua Zheng,[7] Chuanliu Lan,[7] Wencheng Chen,[1] Dan Zhou,[10] and Qisheng Luo[7]

[1]Department of Laboratory Medicine, Affiliated Hospital of Youjiang Medical University for Nationalities, Baise, 533000 Guangxi, China; [2]The Fifth Affiliated Hospital of Guangxi Medical University, Nanning, Guangxi, China; [3]Department of Radiology, Affiliated Hospital of Youjiang Medical University for Nationalities, Baise, 533000 Guangxi, China; [4]Department of Neurology, Guangxi Zhuang Autonomous Region People's Hospital, Nanning, 530021 Guangxi, China; [5]Department of Neurosurgery, People's Hospital of Shenzhen Baoan District, Shenzhen, 518000 Guangdong, China; [6]Department of Neurosurgery, Guangdong 999 Brain Hospital, Guangzhou, 510515 Guangdong, China; [7]Department of Neurosurgery, Affiliated Hospital of Youjiang Medical University for Nationalities, Baise, 533000 Guangxi, China; [8]Department of Pathogenic Biology and Immunology, Youjiang Medical University for Nationalities, Baise, 533000 Guangxi, China; [9]Department of Neurosurgery, Nanfang Hospital, Southern Medical University, Guangzhou, 510515 Guangdong, China; [10]Cancer Center, The First People's Hospital of Foshan, Foshan 528000, China

Glioblastoma has been identified as the most common and aggressive primary brain tumor in adults. Recently, it has been found that cisplatin (DDP) treatment is a common chemotherapeutic method for GBM patients. circ_PTN (ID number: hsa_circ_0003949) is a newly found circular (circRNA) which has been proved to be highly expressed in GBM cells, while its role in GBM remains unclear. Therefore, our study focused on investigating the role of circ_PTN in the DDP resistance of GBM cells. The expression of circ_PTN in DDP-sensitive and DDP-resistant GBM cells was detected in our assay. Functional experiments were utilized to unveil the effects of circ_PTN on the DDP resistance of GBM cells. Moreover, mechanism assays were conducted to confirm the mechanism of how circ_PTN affected the DDP resistance of GBM cells. According to the results, we found that circ_PTN promoted the DDP resistance of GBM cells through activation of the PI3K/AKT pathway. Moreover, circ_PTN silencing inhibited the DDP resistance of GBM tumors *in vivo*. To conclude, our study unveiled the influence of circ_PTN on the DDP resistance of GBM cells, which might provide a therapeutic target for GBM treatment via DDP.

INTRODUCTION

Glioblastoma (GBM) is one of the most common primary aggressive tumors developed in the central nervous system.[1] Although advances have been made in comprehensive therapeutic methods, including surgery, radiotherapy, and chemotherapy, the overall survival of GBM patients remains only near 14 months,[2,3] which is far from favorable. Cisplatin (DDP) is one of the most widely used drugs with curative effect for multiple tumors.[4] Recent studies have suggested that DDP is one of the first-line chemotherapeutic drugs applied for GBM treatment.[5,6] In addition, DDP serves as a DNA damage agent, and its cytotoxic effect is based on the formation of the platinum-DNA complex, which is implicated in the activation of signaling pathways and cell apoptosis.[7] Notably, tumor cells are capable of developing resistance to DDP treatment, which impaired the curative effect of DDP treatment in cancer.[8] Hence, it is necessary to elucidate the mechanisms of how GBM becomes DDP-resistant to find an effective target, thus improving the efficacy of DDP treatment for GBM patients.

Circular RNAs (circRNAs) are a group of non-coding RNAs (ncRNAs) which can play crucial roles in regulating gene expression to affect the progression of various cancers. Recently, accumulating studies have demonstrated that circRNAs can serve as a competing endogenous RNA (ceRNA) to target microRNAs (miRNAs) thus regulating the biological activities of cancer cells.[9] Zhang et al. have proposed that circFOXO3 can upregulate NFAT5 expression to facilitate GBM progression by acting as a ceRNA to target miR-138-5p and miR-432-5p.[10] Moreover, it has been acknowledged that miRNAs

Received 26 May 2020; accepted 31 August 2021;
https://doi.org/10.1016/j.omtn.2021.08.034.

[11]These authors contributed equally

Correspondence: Kinru Wei, Department of Laboratory Medicine, Affiliated Hospital of Youjiang Medical University for Nationalities, Baise, 533000 Guangxi, China
E-mail: chwch3268@sina.com
Correspondence: Zhou Dan, Cancer Cente, The First People's Hospital of Foshan, Foshan 528000, China
E-mail: zdan14@fsyyy.com
Correspondence: Qisheng Luo, Department of Neurosurgery, Affiliated Hospital of Youjiang Medical University for Nationalities, Baise, 533000, Guangxi, China.
E-mail: 850198155@qq.com

This is an open access article under the CC BY license (http://creativecommons.org/licenses/by/4.0/).

are a class of short ncRNAs with the length of 20–23 nucleotides.[11] In addition, such RNAs can exert specific functions through binding to target mRNAs in the 3′ untranslated regions (UTRs), so as to regulate various biological processes, including cell proliferation, migration, and apoptosis.[12] MiR-542-3p functions as a tumor suppressor by targeting mitochondrial rRNA methyltransferase 2 (MRM2; also known as FTSJ2) in the progression of non-small cell lung cancer.[13] However, whether circRNAs can serve as ceRNAs by sponging miRNAs and thus regulating the DDP resistance of GBM cells has not yet been determined.

Signaling pathways, including the phosphoinositide 3-kinase (PI3K)/protein kinase B (AKT) pathway, have been identified to be important participants in the development of cancers.[14] Phosphoinositide-3-kinase regulatory subunit 3 (PIK3R3) is one of the regulatory subunits of PI3K which can stimulate the activation of the PI3K/AKT signaling pathway[15]. Accumulating evidence indicates that the PI3K/AKT signaling pathway is constitutively activated in GBM cells, which has been considered as one effective therapeutic target for GBM.[16,17] According to previous studies, it has been found that the PI3K/AKT signaling pathway can inhibit cell apoptosis and promote cell survival, which is crucial for the modulation of chemo-resistance of cancer cells.[18,19] However, the role of the PI3K/AKT signaling pathway in the DDP resistance of GBM cells remains to be further investigated.

In our study, we mainly investigated the role and mechanism of circ_PTN in regulating the DDP resistance of GBM cells. According to the result, we found that circ_PTN was highly expressed in DDP-resistant GBM cells, and the overexpression of circ_PTN enhanced the resistance of GBM cells to DDP. Moreover, circ_PTN activated PI3K/AKT signaling pathway to enhance the DDP resistance of GBM cells via sequestering miR-542-3p to upregulate PIK3R3. We hoped that our study might serve as a novel insight for the clinic treatment of GBM patients.

RESULTS

circ_PTN expression is highly expressed in DDP-resistant GBM cells

Previously, Song et al. have pointed out that 8 circRNAs (circ_COL1A2, circ_VCAN, circ_PTN, circ_SMO, circ_PLOD2, circ_GLIS3, circ_EPHB4 and circ_CLIP2) are upregulated in GBM cells.[20] To investigate whether those circRNAs influence the DDP resistance of GBM cells, RT-qPCR analyses were applied to examine their expression in parental GBM cells and DDP-resistant GBM cells. As shown in Figure 1A, only circ_PTN (ID number: hsa_circ_0003949) was obviously upregulated in two DDP-resistant GBM cells compared with that in the corresponding parental GBM cells, while no obvious change was found in the expression of other circRNAs in both DDP-resistant cells. To further verify such finding, we detected the expression of circ_PTN in GBM cells with or without DDP resistance. Consistently, the corresponding results manifested that circ_PTN was overexpressed in U87R and U251R cells (Figure 1B). Also, we observed that circ_PTN expression was elevated with the increasing dose of DDP in both DDP-resistant GBM cells (Figure S1A). All these data implied that circ_PTN might affect the DDP resistance of GBM cells. After that, the circular structure of circ_PTN was detected, and the results of Sanger sequencing confirmed the head-to-tail splicing of circ_PTN (Figure 1C). Also, two sets of primers were designed to validate the presence of circ_PTN with divergent primers used to amplify circ_PTN and convergent primers, to amplify linear PTN. As shown by the result of agarose gel electrophoresis revealed that only the linear PTN but not circ_PTN could be amplified using genomic DNA (gDNA); however, with cDNA as templates, the linear PTN amplified by convergent primer was digested by Rnase R, while no obvious change was found in the circ_PTN amplified by divergent primer after Rnase R treatment (Figure 1D). Additionally, we further discovered that under Act D treatment, circ_PTN was more stable than linear PTN, and its half-life was longer than 24 h (Figure 1E). Above results proved that circ_PTN exhibited a circular form in DDP-resistant GBM cells. Simultaneously, subcellular fractionation and FISH assays were conducted to verify the cellular distribution of circ_PTN and we found that such circRNA was mainly distributed in the cytoplasm of DDP-resistant GBM cells (Figures 1F and 1G), suggesting that circ_PTN might function in DDP-resistant GBM cells through post-transcriptional regulation. Totally, circ_PTN is highly expressed in DDP-resistant GBM cells.

Silencing of circ_PTN improves the DDP sensitivity of DDP-resistant GBM cells

To further determine the role of circ_PTN in DDP-resistant GBM cells, functional assays were carried out. First, we stably transfected 3 shRNAs (sh-circ#1, sh-circ#2 and sh-circ#3) targeting different sections of circ_PTN into DDP-resistant GBM cells. Then, RT-qPCR analysis was completed, and it was verified that the transfection of sh-circ#1 and sh-circ#2 successfully silenced the expression of circ_PTN while having no effect on the expression of PTN mRNA. As we found that the transfection of sh-circ#3 led to opposite results, sh-circ#1 and sh-circ#2 were kept for the following assays (Figures 2A and 2B). On this basis, cells transfected with sh-circ#1 or sh-circ#2 were exploited subsequently. As examined by CCK-8 assays, it was found that cell viability was more easily inhibited by increasing the dose of DDP in DDP-resistant GBM cells after the silencing of circ_PTN (Figure 2C), suggesting that DDP-resistant GBM cells with downregulated circ_PTN were more sensitive to DDP treatment. To further examine the impact of circ_PTN on DDP resistance of GBM cells, we then treated DDP-resistant GBM cells with 0 μM or 6 μM DDP to perform loss-of-function assays. The data of the EdU assay indicated that circ_PTN knockdown markedly decreased the number of EdU positive cells in U87R and U251R cells under the treatment of 6 μM DDP, while no significant change was observed in that of the 0 μM DDP group (Figure 2D). Similarly, according to the result of colony formation assays, it was further validated that cell proliferation was apparently hampered by circ_PTN knockdown in DDP-resistant GBM cells treated with 6 μM DDP (Figure 2E). Meanwhile, we also used flow cytometry analysis to detect the effect of circ_PTN on the apoptosis of DDP-resistant GBM cells. As indicated in Figure 2F, the percent of apoptotic cells was increased by

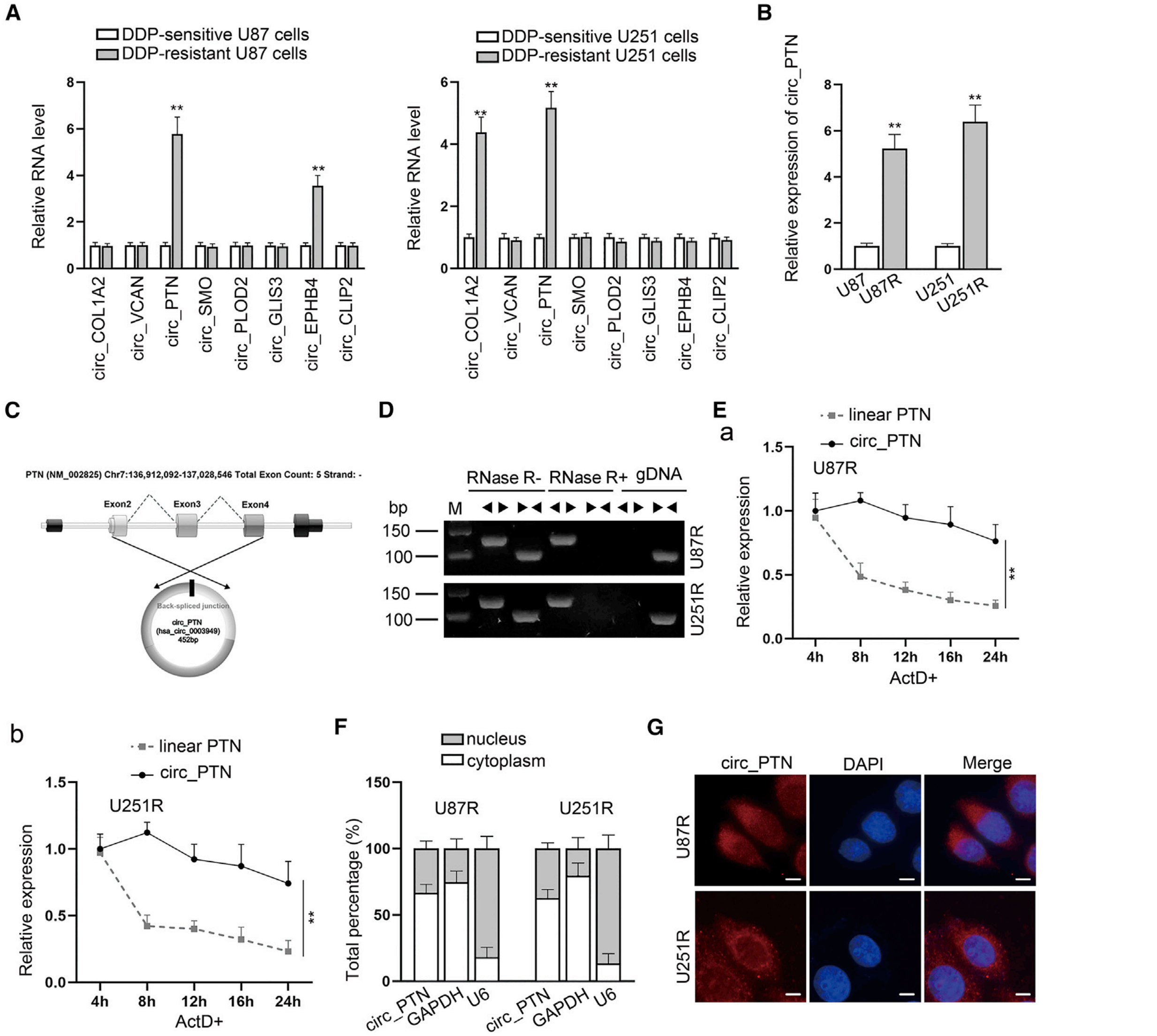

Figure 1. Circ_PTN is highly expressed in DDP-resistant GBM cells

(A) RT-qPCR assay was used to examine the RNA levels of 8 circRNAs in DDP-resistant GBM cells. (B) Circ_PTN expression was measured in DDP-resistant GBM cells. (C) Sanger sequencing was adopted to confirm the head-to-tail splicing of circ_PTN. (D) Rnase R digestion experiment and agarose gel electrophoresis analysis were utilized to detect the existence of circ_PTN in DDP-resistant GBM cells. (E) The level of circular and linear transcripts of PTN in DDP-resistant GBM cells under Act D treatment was assessed. (F)–(G). Subcellular fractionation and FISH assays were conducted to verify the cellular distribution of circ_PTN in DDP-resistant GBM cells. $^{**}p < 0.01$.

circ_PTN silencing in DDP-resistant GBM cells treated with 6 μM DDP whereas no obvious change was observed in DDP-resistant GBM cells without DDP treatment. Furthermore, western blot assay was conducted to examine the level of apoptosis-associated proteins. We found that circ_PTN silencing apparently decreased the protein level of Bcl-2 while elevating the protein level of Cleaved Caspase-3 and Cleaved Caspase-9. However, such phenomena could only be seen in U87R and U251R cells treated with 6 μM DDP (Figure 2G). In addition, results of Transwell assays demonstrated that the silencing of circ_PTN had no evident influence on the migration of U87R and U251R cells without DDP treatment, but obviously hampered the migration of the resistant cells under the treatment of 6 μM DDP (Figure S1B). All those results demonstrates that the silencing of circ_PTN strengthens the DDP sensitivity of DDP-resistant GBM cells.

Overexpression of circ_PTN enhances the resistance of GBM cells to DDP

Our next step was to verify whether the overexpression of circ_PTN affected the DDP resistance of parental GBM cells. First, we upregulated circ_PTN in U87 and U251 cells by transfecting the cells with pcDNA_circ, and adopted RT-qPCR assay to test the transfection

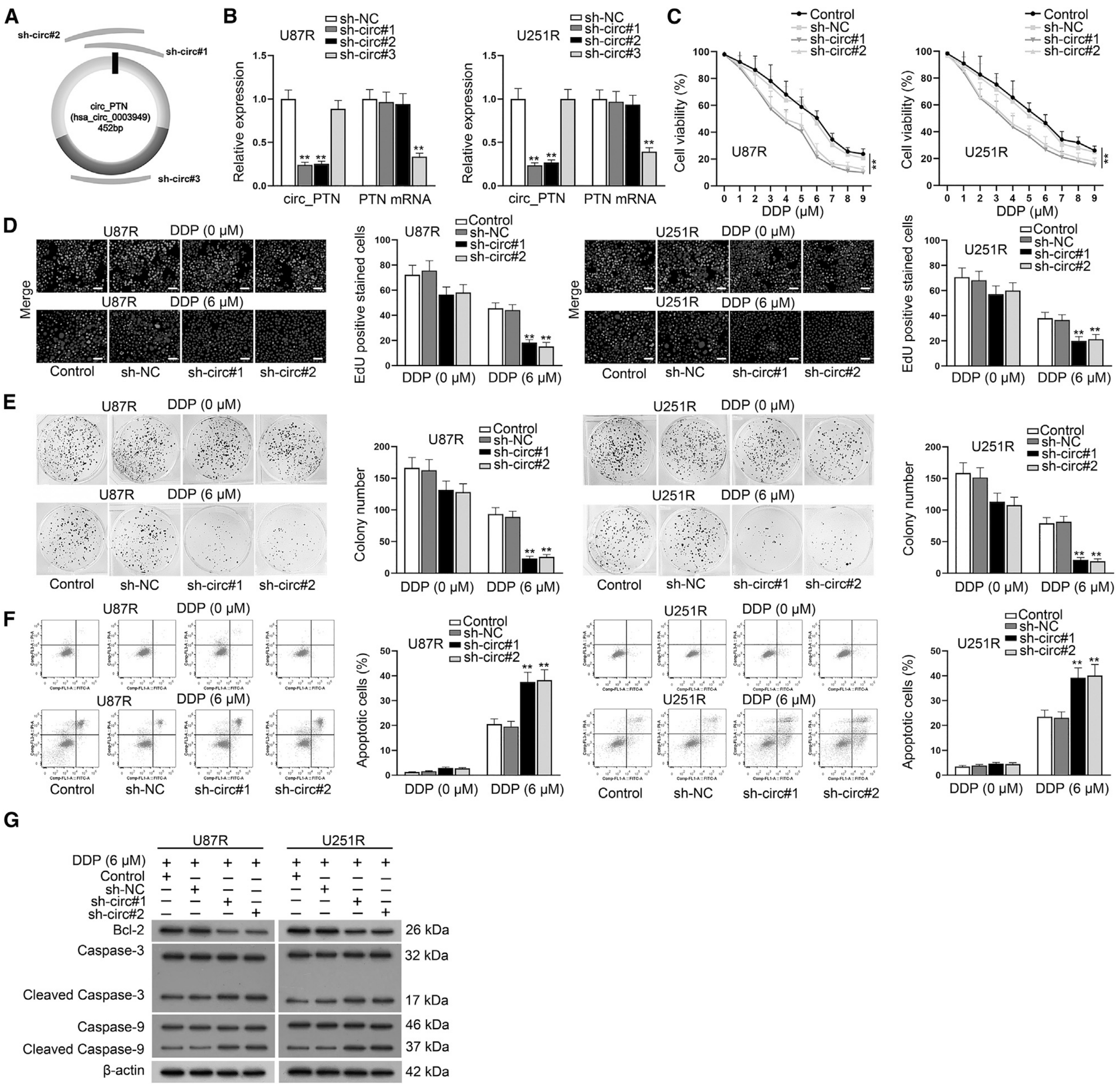

Figure 2. Silenced circ_PTN enhances the DDP sensitivity of DDP-resistant GBM cells

(A) The targeting area of three shRNAs against circ_PTN were presented. (B) RT-qPCR assay was conducted to detect the levels of circ_PTN or PTN mRNA in DDP-resistant GBM cells upon circ_PTN silencing. (C) CCK-8 assay was conducted to detect the viability of DDP-resistant GBM cells under different transfection conditions treated with increasing dose of DDP. (D)–(E) EdU and colony formation assays were utilized to examine the proliferation of DDP-resistant GBM cells treated with 0 or 6 μM DDP upon circ_PTN silencing. (F) Flow cytometry assay was conducted to examine the apoptosis of DDP-resistant GBM cells treated with 0 or 6 μM DDP upon circ_PTN silencing. (G) western blot analysis was conducted to detect the levels of apoptosis-associated proteins related to apoptosis of DDP-resistant GBM cells treated with 0 or 6 μM DDP upon circ_PTN silencing, with β-actin as the loading control. $^{**}p < 0.01$.

efficiency of circ_PTN overexpression (Figures 3A and 3B). Then, a series of gain-of-function assays were conducted. It was foumd that the viability of GBM cells transfected with pcDNA_circ was less inhibited by increasing the dose of DDP more than other groups (Figure 3C), indicating that GBM cells with high circ_PTN expression had elevated resistance to DDP treatment. Consistently, the data from EdU and colony formation assays demonstrated that circ_PTN overexpression augmented the proliferation of GBM cells treated with or without DDP and the facilitating effect on GBM cells treated with 1.5 μM DDP was stronger than those without DDP treatment

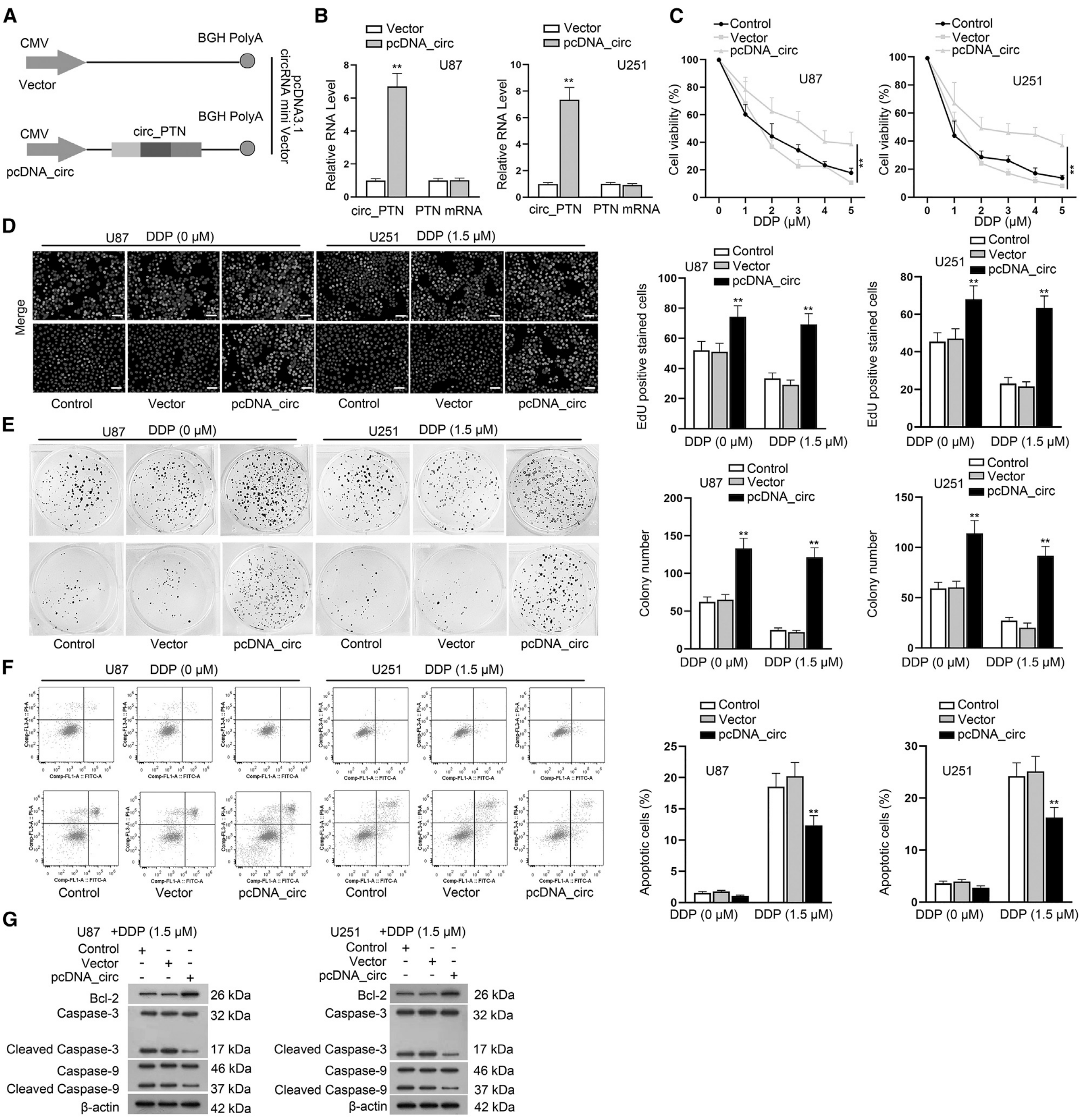

Figure 3. Overexpression of circ_PTN enhances the resistance of GBM cells to DDP

(A) Diagrams of the pcDNA empty vector and the constructs of pcDNA-circ_PTN were presented. (B) The levels of circ_PTN and PTN mRNA in GBM cells upon circ_PTN overexpression were detected. (C) Cell viability of GBM cells transfected with pcDNA_circ treated by indicated dose of DDP was detected by CCK-8 assay. (D)–(E). EdU and colony formation assays were conducted to examine the effect of circ_PTN overexpression on the proliferation of GBM cells treated with 0 or 1.5 μM DDP. (F)–(G). The apoptosis of GBM cells transfected with pcDNA_circ under 0 or 1.5 μM DDP treatment were detected by flow cytometry and western blot assays. **p < 0.01.

(Figures 3D and 3E). In the meantime, the results of flow cytometry analysis indicated that the enhanced apoptosis of GBM cells under 1.5 μM DDP treatment was mitigated evidently by circ_PTN overexpression, while no obvious change was caused by circ_PTN overexpression to cells without DDP treatment (Figure 3F). Additionally, the data from western blot assay also demonstrated that the protein level of Bcl-2 was increased while that of Cleaved Caspase-3 and Cleaved Caspase-9 was decreased in circ_PTN-overexpressed GBM

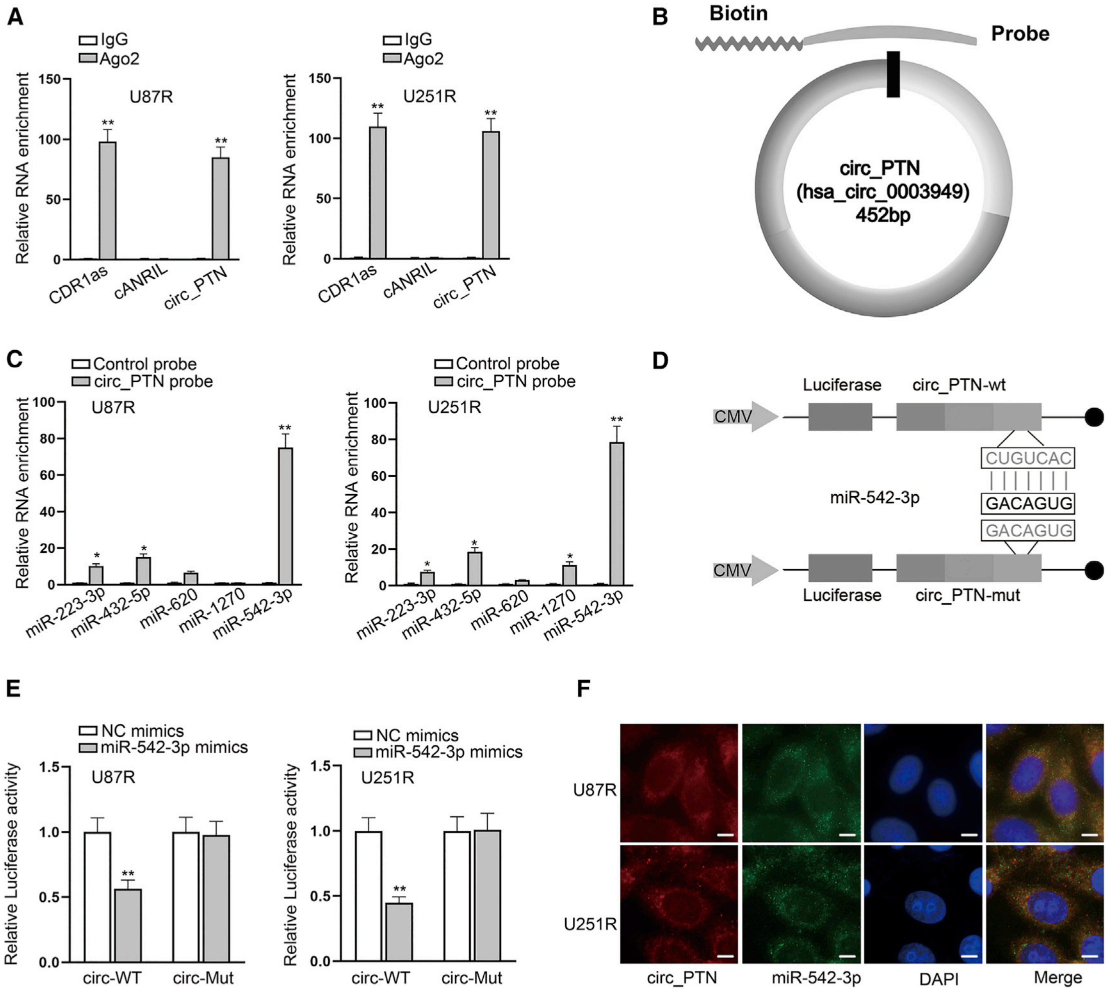

Figure 4. MiR-542-3p is the downstream gene of circ_PTN

(A) RIP assays were used to detect the enrichment of CDR1as, cANRIL and circ_PTN in Ago2 groups. (B) A schematic diagram demonstrating the design of a biotin-labeled circ_PTN probe for RNA pull down assay was presented. (C) RNA pull down assays were utilized to detect the enrichment of miRNAs in circ_PTN probe groups. (D) A schematic illustration showing the luciferase reporters containing circ_PTN-WT or circ_PTN-Mut with wild-type or mutant miR-542-3p binding sites was presented. (E) Luciferase reporter assays were conducted to verify the binding relationship between circ_PTN and miR-542-3p. (F) FISH assays were carried out to determine the co-localization of circ_PTN and miR-542-3p in DDP-resistant GBM cells. $^*p < 0.05$, $^{**}p < 0.01$.

cells treated with 1.5 μM DDP (Figure 3G). Moreover, results of Transwell assay indicated that upregulation of circ_PTN promoted GBM cell migration, and it also effectively alleviated the suppression of DDP treatment on GBM cell migration (Figure S1C). Collectively, circ_PTN overexpression enhances the resistance of GBM cells to DDP.

circ_PTN acts as a ceRNA to sequester miR-542-3p

Since we had proved that circ_PTN was mainly located in the cytoplasm of DDP-resistant GBM cells, we speculated that circ_PTN may exert its functions through a ceRNA mechanism. Therefore, we first used RIP assay to confirm the association between circ_PTN and Ago2. According to one previous study, CDR1as has been reported to be a ceRNA to target miRNA during the progression of cancers while cANRIL cannot.[21] Here we used CDR1as as the positive control and cANRIL as the negative control. As revealed in Figures 4A and S2A, circ_PTN and CDR1as were highly enriched in Ago2 groups, which was in line with our expectation. Then we consulted the StarBase Data: http://starbase.sysu.edu.cn to predict the potential miRNAs that may interact with circ_PTN, and five candidates (hsa-miR-223-3p, hsa-miR-432-5p, hsa-miR-620, hsa-miR-1270, and hsa-miR-542-3p) were selected (Figure S2B). Thereafter, we designed a biotin-labeled circ_PTN probe targeting the splicing junction for the RNA pull down assay (Figure 4B). As shown by the result of the RNA pull down assay, we found that miR-542-3p was most abundantly pulled down by the circ_PTN probe among the five miRNA candidates in both DDP-resistant U87 and U251 cells (Figures 4C and S2C). Besides, it

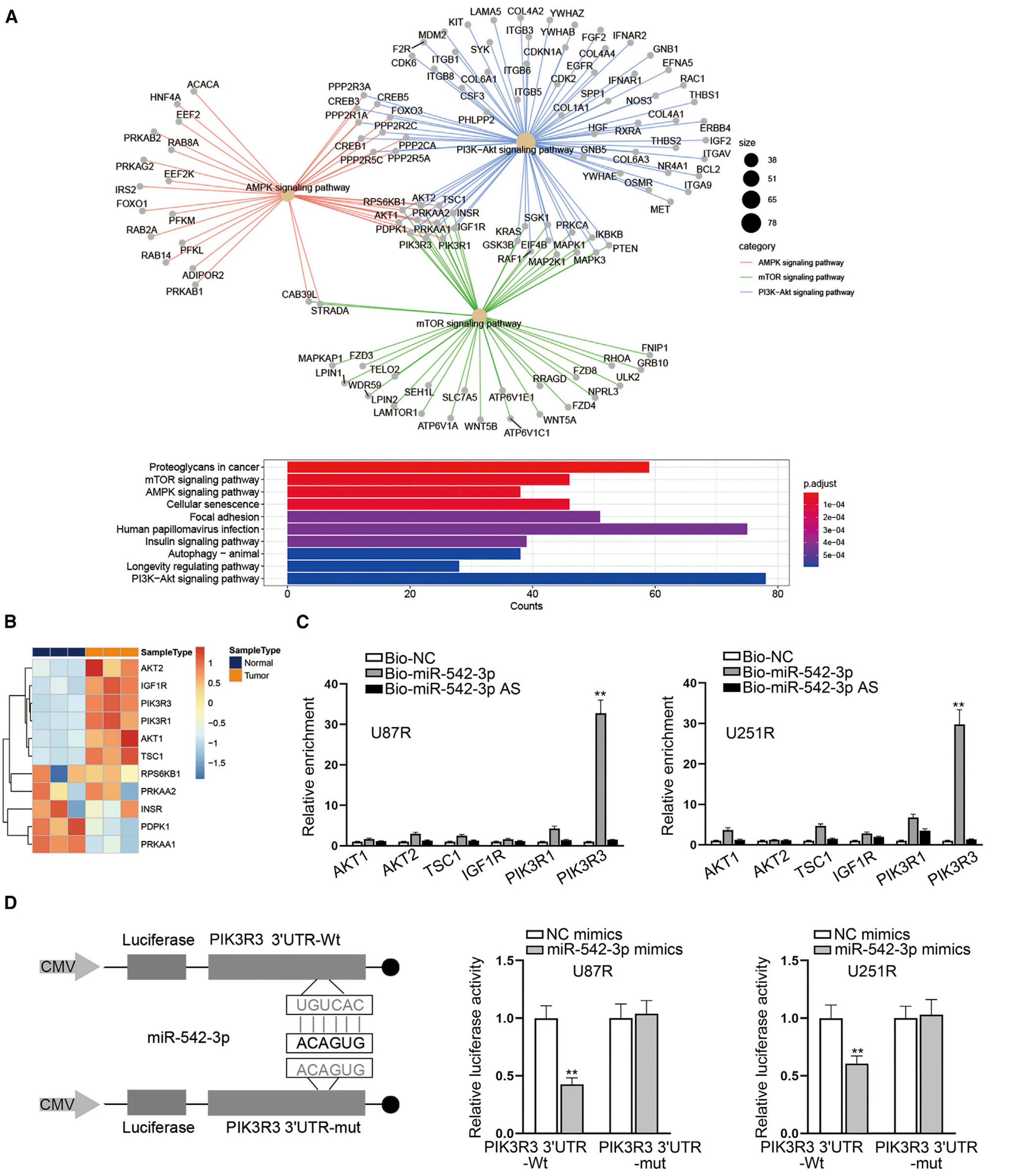

(legend on next page)

was proved that the miR-542-3p expression was dramatically downregulated in U87R and U251R cells relative to the parental cells (Figure S2D). Hence, miR-542-3p was kept in the following studies. Before investigating the relationship between miR-542-3p and circ_PTN, we constructed luciferase reporters covering circ_PTN-WT/Mut with wild-type or mutant miR-542-3p binding sites (Figure 4D) and then overexpressed miR-542-3p in both parental and DDP-resistant GBM cells (Figures S2E and S2F). The results of luciferase reporter assay showed that the overexpression of miR-542-3p led to a marked decrease in the luciferase activity of circ_PTN-WT reporters while no effects on that of circ_PTN-Mut reporters were observed (Figure 4E). In addition, data from FISH assays further validated the co-localization of circ_PTN and miR-542-3p in the cytoplasm of U87R and U251R cells (Figure 4F). To conclude, circ_PTN interacts with miR-542-3p in DDP-resistant GBM cells.

PIK3R3 is the target gene of miR-542-3p

According to previous studies, miRNAs are implicated in multiple biological processes of various diseases via silencing their target mRNAs. Thus, deciphering miRNA targets is of great importance to the diagnosis and treatment of diseases.[22] Here, we analyzed the signaling pathways affected by the downstream genes of miR-542-3p and screened the top 10 signaling pathways. According to the result, we found that three signaling pathways (AMPK, PI3K/AKT, and mTOR) were related to DDP resistance among those signaling pathways (Figure 5A). Then we filtered the intersection gene of three pathways and conducted RT-qPCR analysis. As illustrated in Figure 5B, the fold changes of AKT2, AKT1, TSC1, IGF1R, PIK3R1, and PIK3R3 were the highest. To further confirm which mRNA was targeted by miR-542-3p in DDP-resistant GBM cells, RNA pull-down assays were carried out. As shown by the results, it was indicated that PIK3R3 was the most obviously enriched one of the Bio-miR-542-3p groups in both U87R and U251R cells (Figure 5C). As PIK3R3 has been proved to be the receptor of PI3K/AKT signaling pathway, we speculated that miR-542-3p might regulate the PI3K/AKT signaling pathway through targeting PIK3R3. Therefore, we selected PIK3R3 for further verification. Through RIP assays, we found that PIK3R3 and miR-542-3p were both precipitated by Ago2 (Figure S2G), suggesting that PIK3R3 and miR-542-3p could co-exist in Ago2-related RNA-induced silencing complex (RISC). Moreover, we performed luciferase reporter assays to corroborate the relationship between PIK3R3 and miR-542-3p. As expected, the luciferase activity was significantly decreased in DDP-resistant GBM cells co-transfected with PIK3R3 3′UTR-Wt and miR-542-3p mimics while no statistical significance of that was observed in DDP-resistant GBM cells co-transfected with PIK3R3 3′UTR-Mut and miR-542-3p mimics (Figure 5D). In short, PIK3R3 is targeted by miR-542-3p in DDP-resistant GBM cells.

miR-542-3p inhibits the DDP resistance by targeting PIK3R3

To further elucidate the role of PIK3R3 in DDP resistance, we analyzed its expression in the parental and DDP-resistant GBM cells. As manifested in Figure 6A, the mRNA and protein levels of PIK3R3 were considerably upregulated in DDP-resistant GBM cells compared to the control groups. Then we overexpressed PIK3R3 in U87R and U251R cells (Figure S2H), and adopted RT-qPCR and western blot assays to further detect the relationship between PIK3R3 and miR-542-3p. It was found that the mRNA and protein levels of PIK3R3 which declined by miR-542-3p overexpression could be significantly recovered upon PIK3R3 overexpression (Figure 6B). Subsequently, a series of rescue experiments were conducted in DDP-resistant GBM cells treated with 6 μM DDP. As expected, the result of CCK-8 assays showed that the viability impaired by miR-542-3p overexpression could be greatly reversed by the co-transfection of pcDNA_PIK3R3 (Figure 6C). Consistently, by conducting EdU assays, we observed that the upregulation of PIK3R3 could partially reverse the reducing effect of miR-542-3p overexpression on EdU positive stained cells (Figures 6D and S3A). Likewise, colony formation assay data suggested that the reduced number of colonies caused by miR-542-3p overexpression was obviously recovered by the co-transfection of pcDNA_PIK3R3 (Figure 6E). In addition, the results from flow cytometry analysis indicated that PIK3R3 upregulation counteracted the suppressive effect caused by miR-542-3p overexpression on cell apoptosis (Figure 6F). Finally, western blot assay was conducted and we observed that the decreased protein level of Bcl-2 and the elevated protein levels Cleaved Caspase-3 and Cleaved Caspase-9 caused by miR-542-3p mimics were significantly reversed after the co-transfection of pcDNA_PIK3R3 (Figures 6G and S3B). Also, the repressive impact of miR-542-3p upregulation on cell migration was significantly offset upon co-overexpression of PIK3R3 (Figure S3C). Taken together, miR-542-3p attenuates the DDP resistance of DDP-resistant GBM cells by targeting PIK3R3.

circ_PTN promotes the DDP resistance of GBM cells by regulating miR-542-3p and PIK3R3

After confirming that miR-542-3p attenuated the DDP resistance of DDP-resistant GBM cells by targeting PIK3R3, our next step was to investigate the influence of the circ_PTN/miR-542-3p/PIK3R3 axis on GBM cells treated with 6 μM DDP. First, we silenced the expression of miR-542-3p in DDP-resistant GBM cells by using miR-542-3p inhibitor (Figure S3D). Then we proved that miR-542-3p inhibition could significantly reverse the downregulation of PIK3R3 induced by circ_PTN deficiency in DDP-resistant GBM cells (Figures 7A and S3E). Similarly, miR-542-3p overexpression attenuated the

Figure 5. PIK3R3 is directly targeted by miR-542-3p

(A) An analysis of the pathways under the downstream targets of miR-542-3p, and microarray data of top 10 signaling pathways in DDP-resistant GBM cells were presented. (B) A heatmap showing the RT-qPCR analysis of the intersection genes in three signaling pathways was presented. (C) RNA pull-down assays were conducted to examine the binding relationship between certain genes and miR-542-3p. (D) A schematic illustration showing the luciferase reporters containing PIK3R3 3′UTR-WT or PIK3R3 3′UTR-Mut was presented and luciferase reporter assays were conducted to detect the binding relationship between miR-542-3p and PIK3R3. $^{**}p < 0.01$.

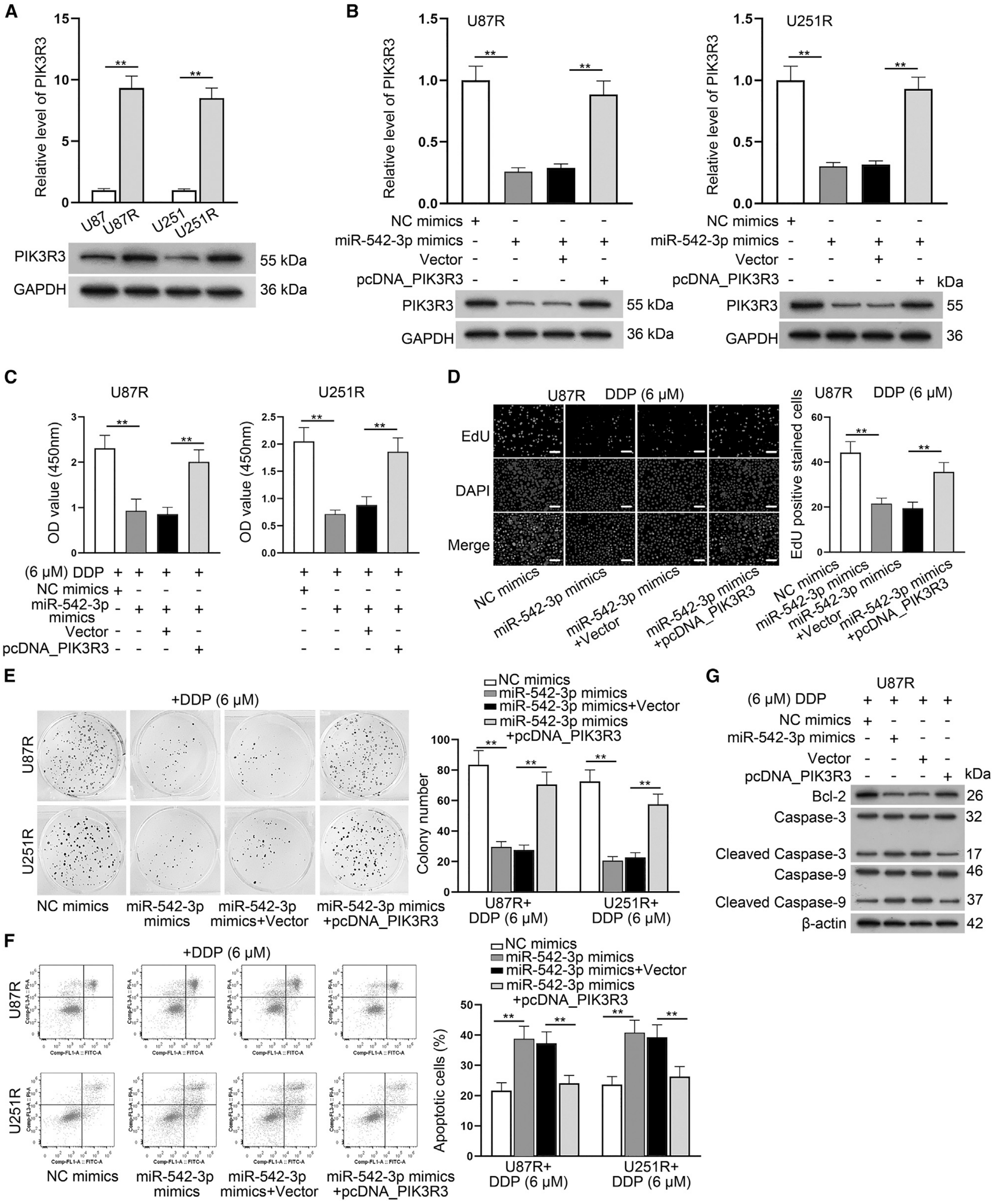

(legend on next page)

upregulated expression of PIK3R3 due to circ_PTN overexpression in DDP-resistant GBM cells (Figures 7B and S3F). Then a series of rescue experiments were carried out. It was indicated by the results of CCK-8 and EdU assays that the co-transfection of miR-542-3p inhibitor or pcDNA_PIK3R3 could greatly reverse the inhibition on cell proliferation mediated by circ_PTN knockdown (Figures 7C–7F and S3G–S3J). Similarly, the results of colony formation assays further supported that cell proliferation hampered by circ_PTN depletion was rescued by the co-transfection of miR-542-3p inhibitor or pcDNA_PIK3R3 (Figures 7G, 7H, S4A, and S4B). In addition, the result of flow cytometry analysis indicated that cell apoptosis enhanced by circ_PTN knockdown was greatly reversed upon miR-542-3p inhibition or PIK3R3 overexpression (Figures 7I and S4C). Simultaneously, circ_PTN knockdown-induced elevation in the protein levels of Cleaved Caspase-3 and Cleaved Caspase-9 was significantly counteracted by miR-542-3p inhibition or PIK3R3 overexpression (Figure S4D). Likewise, the mitigated cell migration caused by circ_PTN deficiency was also recovered upon the inhibition of miR-542-3p or the upregulation of PIK3R3 (Figure S5A). All of the data suggested that circ_PTN contributes to the DDP resistance of DDP-resistant GBM cells by regulating miR-542-3p and PIK3R3.

circ_PTN activates the PI3K/AKT pathway in DDP-resistant GBM cells and it strengthens the DDP resistance of GBM cells *in vivo*

Based on the result that PIK3R3 was the receptor of the PI3K/AKT signaling pathway, we further analyzed the influence of circ_PTN/miR-542-3p/PIK3R3 network on the PI3K/AKT signaling pathway in GBM cells. The results of western blot indicated that silencing of circ_PTN significantly reduced the protein level of PI3K-alpha and p-AKT, while inhibiting miR-542-3p or overexpressing PIK3R3 could greatly recover such reduction in DDP-resistant GBM cells (Figure 8A). Meanwhile, upregulation of circ_PTN significantly increased the expression of PI3K-alpha and p-AKT, and the overexpression of miR-542-3p could greatly abrogate the above effects in DDP-sensitive GBM cells (Figure 8B). Furthermore, by conducting western blot assays, we found that the impact of circ_PTN overexpression on PI3K-alpha, p-AKT and Cleaved Caspase-3 in DDP-sensitive GBM cells was countervailed by the depletion of PIK3R3 or the treatment of PI-103 (a specific PI3K/AKT inhibitor) (Figure 8C and S5B). More importantly, to investigate whether circ_PTN could affect the DDP sensitivity of GBM cells *in vivo*, we subcutaneously injected U87R cells with or without stable circ_PTN knockdown into mice, and the mice were then treated with PBS or 6 μM DDP. As anticipated, the growth rate of tumors from control U87R cells was slightly slowed down while that of tumors from U87R cells with inhibited circ_PTN was greatly reduced under DDP treatment (Figure 8D). In addition, the volume and weight of tumors derived from circ_PTN-depleted U87R cells were significantly decreased after DDP treatment (Figures 8E and 8F). All in all, we concluded that circ_PTN enhances the DDP resistance of GBM cells *in vitro* and *in vivo* through activating the PI3K/AKT pathway by targeting miR-542-3p/PIK3R3 signaling.

DISCUSSION

DDP treatment is one of the most central chemotherapeutic methods for patients with GBM.[23] However, DDP resistance is also a headache in GBM treatment.[24] In recent years, a number of studies have indicated that non-coding RNAs play significant roles in regulating the DDP resistance of GBM cells.[24] Ma et al. have put forward that AC023115.3 has an inhibitory effect on DDP resistance in GBM.[25] Additionally, miR-186 overturns DDP resistance by degrading YY1 in GBM.[26] However, the roles of circRNAs in the DDP resistance of GBM cells have been poorly documented. Previous study has proved that circ_PTN expression was enhanced in GBM cells.[27] Intriguingly, our study further found that circ_PTN was highly expressed in DDP-resistant GBM cells compared to the parental GBM cells. Moreover, we applied loss-of-function and gain-of-function assays and discovered that circ_PTN enhanced the DDP resistance of GBM cells, suggesting that circ_PTN might be a potential target for defeating the DDP resistance of GBM cells.

It has been reported that circ_PTN can affect the function of glioma cells through targeting miR-122/SOX6 or miR-432-5p/RAB10 axis.[27,28] Moreover, Chen et al. have found that circ_PTN promotes the proliferation and stemness of glioma cells via sponging miR-145-5p/miR-330-5p.[29] These literatures have suggested the ceRNA regulation mechanism of circ_PTN in glioma. Likewise, our study pointed out that circ_PTN could interact with miR-542-3p in DDP-resistant GBM cells, while miR-542-3p was downregulated in DDP-resistant GBM cells. As reported previously, miR-542-3p impedes the progression of colorectal cancer by targeting OTUB1.[30] MiR-542-3p suppresses the tumorigenesis and development of ovarian cancer via regulating CDK14.[31] More importantly, miR-542-3p targets the 3′-UTR of Survivin mRNA to overcome HER3-mediated paclitaxel resistance in breast cancer.[32] Furthermore, our study demonstrated that PIK3R3 was the target of miR-542-3p, and PIK3R3 expression was obviously increased in DDP-resistant GBM cells. PIK3R3 has been elucidated to be highly expressed in drug-resistant hepatocellular cancer cells, which is in accordance with our findings.[33]

Notably, PIK3R3 can trigger the activation of the PI3K/AKT signaling pathway.[34] Further, activation of the PI3K/AKT signaling pathway contributes to the DDP resistance of breast cancer cells.[35] MALAT1

Figure 6. MiR-542-3p inhibits the DDP resistance of DDP-resistant GBM cells by targeting PIK3R3

(A) RT-qPCR and western blot assays were conducted to detect the mRNA and protein levels of PIK3R3 in DDP-resistant GBM cells. (B) RT-qPCR and western blot assays were utilized to detect the mRNA and protein levels of PIK3R3 in DDP-resistant GBM cells under different conditions. (C) CCK-8 assay was conducted to detect the viability of indicated DDP-resistant GBM cells upon DDP (6 μM) treatment. (D)–(E). EdU and colony formation assays were utilized to detect the proliferation of DDP-resistant U87R cells under different transfections upon DDP (6 μM) treatment. (F-G) Flow cytometry and western blot assays were conducted to detect the apoptosis of DDP-resistant GBM cells with indicated transfections upon DDP (6 μM) treatment. **$p < 0.01$.

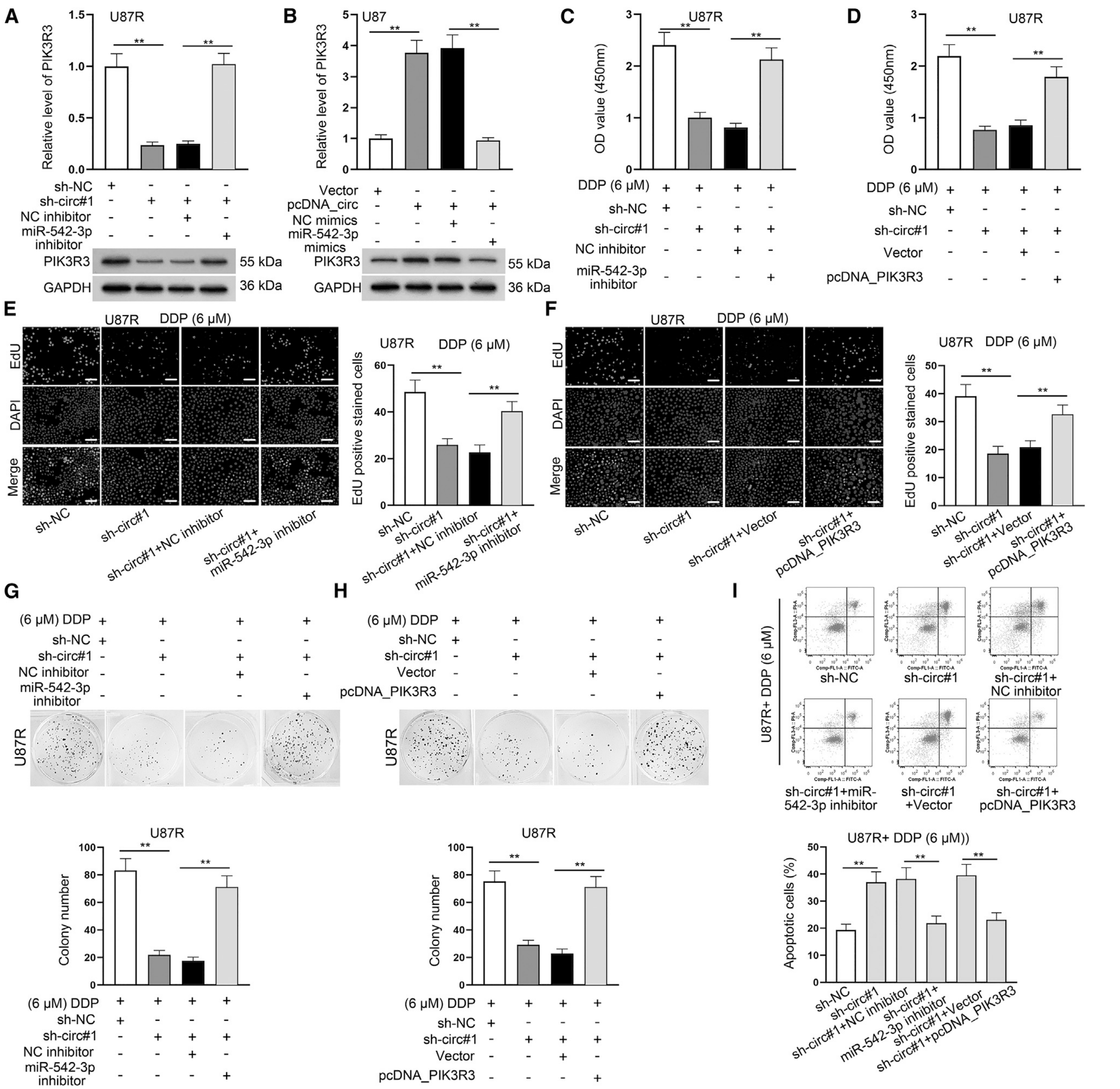

Figure 7. Circ_PTN promotes the DDP resistance by regulating miR-542-3p and PIK3R3

(A) RT-qPCR and western blot assays were conducted to detect the mRNA level and protein level of PIK3R3 in indicated DDP-resistant U87R cells. (B) RT-qPCR and western blot assays were utilized to detect the mRNA and protein levels of PIK3R3 in indicated U87 cells. (C)–(D) CCK-8 assay was carried out to examine the viability of DDP-treated U87R cells under different transfection conditions. (E)–(H) EdU and colony formation assays were utilized to examine the proliferation of DDP-treated U87R cells under different transfection conditions. (I) Flow cytometry assay was conducted to examine the apoptosis rate of DDP-treated U87R cells under indicated transfection conditions. **p < 0.01.

promotes DDP resistance in cervical cancer through activation of the PI3K/AKT pathway.[36]. Cai et al. have proposed that miR-542-3p can target PIK3R1 to activate the AKT signaling pathway in GBM.[37] Also, a former study indicated that circAKT3 enhances DDP resistance in gastric cancer via the activation of PI3K/AKT signaling.[38] Consistent with these findings, we discovered that circ_PTN/miR-542-3p signaling enhanced PIK3R3 to activate the PI3K/AKT signaling pathway, so as to facilitated DDP resistance in GBM cells. Previously, multiple reports have proved that the PI3K/AKT pathway can mediate cell autophagy to enhance the chemo-sensitivity of cancer cells.[39] For example, CD133 activates PI3K/AKT/mTOR signaling to induce autophagy, thus facilitating the DDP resistance of Cis-KATO-III cells.[40]

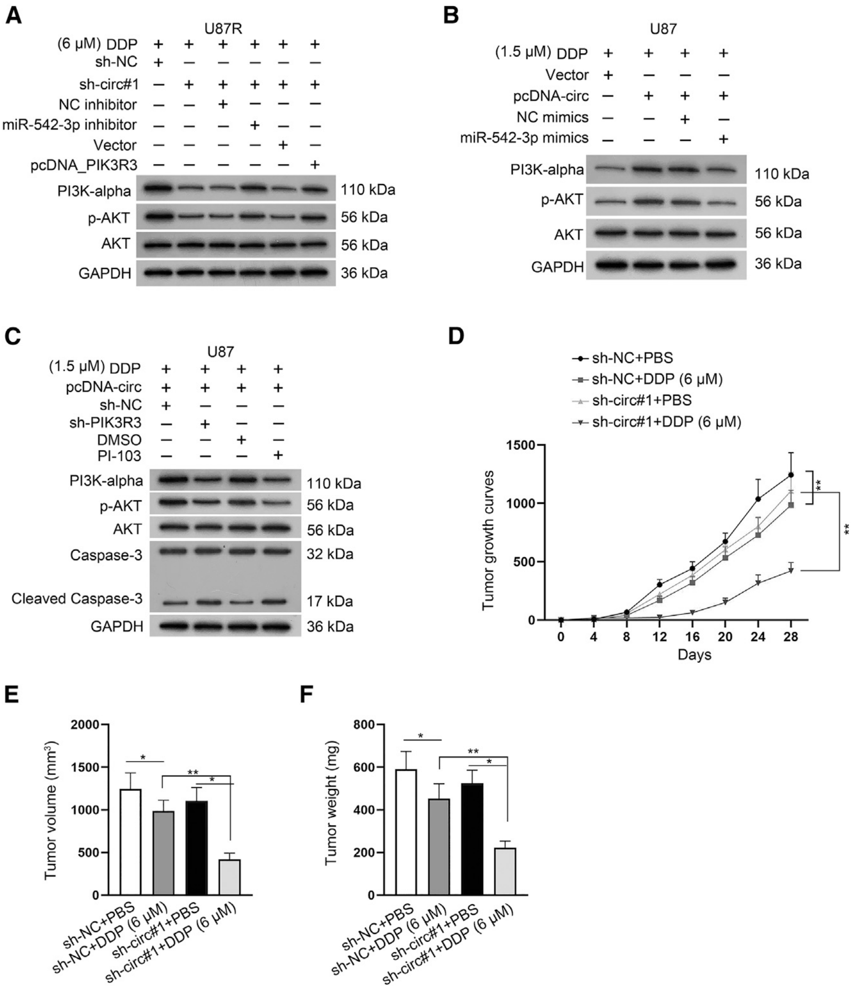

Figure 8. Circ_PTN activates PI3K/AKT pathway in GBM cells and it contributes to DDP resistance of GBM tumors *in vivo*

(A)–(B). Western blot assay was conducted to examine the protein levels of PI3K-alpha, p-AKT and AKT in DDP-treated GBM cells (under different doses) with different transfections. (C) Western blot assay was carried out to examine the levels of certain proteins in DDP-treated GBM cells under different transfections or treatments. (D) The volume of xenograft tumors in mice were measured after the injection of indicated DDP-resistant GBM cells. (E)–(F). The final volume and weight of subcutaneous xenograft tumors excised from mice of indicated groups were presented. *$p < 0.05$, **$p < 0.01$.

Based on this, we could deduce that circ_PTN/miR-542-3p/PIK3R3 axis stimulated PI3K/AKT pathway to regulate autophagy, thus facilitating the DDP resistance of GBM cells.

Conclusively, our study revealed the role and regulatory mechanism of circ_PTN in the DDP resistance of GBM cells, and these findings might provide practical value for improving the efficacy of DDP treatment for GBM patients. However, there are some limitations in the present study: The downstream mechanism of PI3K/AKT signaling pathway in GBM cells has not been identified; also, the upstream regulatory mechanisms of circ_PTN have not been discussed; significantly, the clinical value of circ_PTN in GBM has not been analyzed; for the *in vivo* experiments, we have not totally simulated the process of drug resistance in the present work. These drawbacks will be further improved in follow-up studies.

MATERIALS AND METHODS

Cell culture

The DDP-sensitive GBM cell lines (U87 and U251) and the immortalized human embryo kidney cell line 293T were purchased from the Chinese academy of sciences cell bank (Shanghai, China). The DDP-resistant GBM cell strains (U87R and U251R) were developed from the parental U87 and U251 cells through exposing them to gradually increasing dose of DDP as described.[41] Cells were all cultured in DMEM medium (Invitrogen, Carlsbad, CA, USA) adding 10% fetal bovine serum (FBS, Invitrogen).

circRNA confirmation and quantitative reverse transcription real-time polymerase chain reaction (RT-qPCR)

Total RNA was extracted from cells with TRIzol reagent (Invitrogen, Carlsbad, CA, USA) based on the manufacturer's instruction. RNA (with/without RNase R treatment) was then reversely transcribed and analyzed via RT-qPCR using a SYBR-Green PCR kit (Invitrogen). Divergent and convergent primers were used for the validation of circular or linear transcripts of PTN. The experiments were conducted in triplicate.

Actinomycin D (Act D) experiment

U87R and U251R cells were seeded in 24-well plates (5×10^4/well). After 24 h, cells were treated with Act D (2 μg/ml, Abcam, Cambridge, MA, USA) for 4, 8, 12, 16, and 24 h. After digestion for the indicated times, the relative expression of circ_PTN and linear PTN was analyzed by RT-qPCR. The experiments were performed in triplicate.

Subcellular fractionation assay

The fractionation of cell nucleus and cytoplasm was performed via a PARIS Kit (Invitrogen) in line with the manufacturer's requirements. GAPDH and U6 were employed for the control of cytoplasmic and nuclear RNA, respectively. Finally, circ_PTN in cytoplasm/nuclear was examined by qRT-PCR. The experiments were carried out in triplicate.

Fluorescence *in situ* hybridization (FISH) assay

To study the location of circ_PTN and miR-542-3p, FISH assays were performed using Fluorescent *in Situ* Hybridization Kits (RiboBio,

Guangzhou, China) according to the manufacturer's requirements. DAPI was used to re-stain the nuclei. A laser-scanning, confocal microscope was then used to observe the stained cells. GAPDH and U6 were treated as the cytoplasm and the nucleus control, respectively. The experiments were conducted in triplicate.

Cell transfection

Short hairpin RNAs (shRNAs) targeted the back-splice junction sites of circ_PTN (sh-circ#1/2/3) and sh-NC. Besides, miR-542-3p mimics/inhibitor and the corresponding negative controls (NC mimics/inhibitor) were cloned and synthesized by GenePharma (Shanghai, China). Also, pcDNA vectors loaded with circ_PTN or PIK3R3 were applied for overexpression, with the empty vector as negative control. Transfections of indicated plasmids into GBM cells lasted -48 h using lipofectamine 3000 (Thermo Fisher, USA). Expression levels of the genes were determined with RT-qPCR. The experiments were conducted in triplicate.

RNA immunoprecipitation (RIP) assay

RIP assays were conducted with a Magna RIP Kit (Millipore, Billerica, MA, USA) following the manufacturer's protocols. Cells were lysed with RNA lysis buffer. Then, cell lysates were incubated with magnetic beads conjugated to anti-Argonaute2 (Ago2) or anti-IgG antibody at 4°C for 4 h. Next, the beads were washed and the immunoprecipitated complex was purified and detected by RT-qPCR analysis. The experiments were conducted in triplicate.

RNA pull-down assay

Biotin-labeled circ_PTN or miR-542-3p probes were designed and synthesized by GenePharma (Shanghai, China). Cells were lysed with lysis buffer and then the lysates were incubated with specific biotin-labeled probes for 2 h. Then the mixtures were incubated with streptavidin-coated magnetic beads to pull down the biotin-labeled RNA complex for another 4 h. After washing, the RNA complex was extracted with TRIzol (Takara, Dalian, China) and then detected by RT-qPCR analysis. The experiments were performed in triplicate.

Luciferase reporter assay

Circ_PTN sequences (or PIK3R3-3′UTR sequences) containing wild-type or mutated-type of miR-542-3p binding sites were synthesized and separately inserted into pmirGLO luciferase reporter vectors (Promega, Madison, WI). Cells were seeded into 24-well plates at a density of 3×10^4 cells per well. After co-transfection with miR-542-3p mimics or NC mimics and the above constructed luciferase plasmids for 48 h, the luciferase activity was measured through a dual-luciferase reporter assay system (Promega). The experiments were conducted in triplicate.

Cell counting kit-8 (CCK-8) assay

After transfection, cells (2×10^4 cells/well) were seeded into 96-well plates and then placed in an incubator with 5% CO_2 at 37°C for 24 h. Thereafter, 10 μL of CCK-8 reagent was added to each well for 4 h of incubation. The absorbance values at a wavelength of 450 nm were measured using a microplate reader (Bio-Tek Instruments, Hopkinton, MA, USA) to evaluate the viability of indicated cells. The experiments were conducted in triplicate.

Colony formation assay

The cells (800 cells/well) were seeded into 6-well plates for 2 weeks of incubation. The medium was changed every 3 days. Then, the plates were fixed with 4% paraformaldehyde and stained with 0.1% crystal violet. After that, colonies with no less than 50 cells were subjected to counting manually. Triplicate is required for each experiment.

5-Ethynyl-2′-deoxyuridine (EdU) assay

Proliferation ability of indicated GBM cells was evaluated using an EdU Detection Kit (RiboBio, Guangzhou, China) according to the manufacturer's claims. After transfections, cells were cultured in 96-well plates at a density of 1×10^4 cells per well. Then, 50 μL EdU labeling medium was added to the plates, which were incubated for 2 h at 37°C with 5% CO_2. After fixation with 4% paraformaldehyde and permeation via 0.5% Triton X-100 for 10 min, cells were incubated for 30 min with 100 μL 1 × Apollo®488 fluorescent dry reaction solution under 37°C. Nuclei were stained with DAPI. The percent of EdU positive cells was calculated under a fluorescence microscope (Shanghai, China). Each experiment was conducted three times.

Flow cytometry analysis

The 2×10^5 cells during logarithmic phase were seeded into 6-well plates. After 24 h of cell culture, DDP was added into each well and then the plates were cultivated for 48 h at 37°C under 5% CO_2 and saturated humidity conditions. Next, cells were collected and washed. Then, cells were subjected to staining using an Annexin-V-FITC detection kit (BioVision, San Francisco, CA, USA), and the apoptosis rate was examined through flow cytometry (BD Biosciences, Franklin Lake, NJ, USA). Each experiment was conducted three times.

Western blot analysis

Proteins were extracted from cells using RIPA buffer (CWBIO, Beijing, China). After being electrophoresed by SDS-PAGE, the protein samples were transferred to membranes and incubated with specific primary antibodies at 4°C overnight. Then the membranes were incubated with secondary antibodies at room temperature for 1 h. The protein bands were visualized using enhanced chemiluminescent (ECL) agent (Millipore, Germany). The primary antibodies were as follows: Bcl-2, Caspase-3, Cleaved Caspase-3, Caspase-9, Cleaved Caspase-9, β-actin, PIK3R3, GAPDH, PI3K-alpha, p-AKT and AKT. The details of primary and secondary antibodies were provided in Table S1. The experiments were performed in triplicate.

Transwell assay

Cell migration was assessed by use of 24-well transwell inserts (BD Biosciences). In detail, 1×10^5 cells were supplemented into the upper chamber and 500 μL of complete culture medium was filled into the lower chamber. After 24 h of incubation, the migrated cells were fixed and stained by 4% paraformaldehyde and 0.1% crystal violet, respectively. After that, cells in random five fields were observed under a

light microscope (Olympus, Tokyo, Japan). The experiment was performed in triplicate.

Mouse xenograft model

BALB/c (4-week-old) nude mice were purchased from Kay Biological Technology Co., Ltd. (Shanghai, China). U87R cells (1×10^6) transfected with sh-circ#1 or sh-NC were subcutaneously injected into 6 mice, respectively. Then, three random mice from each group were treated with PBS, while the other three were treated with 6 μM DDP. Four weeks after injection, the mice were sacrificed, and tumors were removed to be weighed and the tumor size was measured. The animal experiment protocol was approved by the Animal Experimentation Ethics Committee of Affiliated Hospital of Youjiang Medical University for Nationalities.

Gene accession numbers

Accession numbers of the genes involved in our study can be found in Table S2.

Statistical analysis

All data from experiments including the three biological replications were exhibited as the mean ± standard deviation (SD). Deviation comparisons were performed using Student's t test or one-way ANOVA. The statistical significance in differences was confirmed when $p < 0.05$. GraphPad Prism 5.0 software (La Jolla, CA, USA) was used for the statistical analysis. All experiments were performed in triplicate.

SUPPLEMENTAL INFORMATION

Supplemental information can be found online at https://doi.org/10.1016/j.omtn.2021.08.034.

ACKNOWLEDGMENT

Thanks a lot for the support of laboratory. H.L., T.Y., D.H., and X.C. contributed to project administration, study design and data curation. X.L., Q.W., Haineng Huang, Huadong Huang, and H.W. were responsible for investigation and methods. J.W., Q.T., and Q.L. drafted the manuscript. The others performed experiments. All authors have read and approved the final manuscript. This work was supported by the National Natural Science Foundation of China, Grant/Award Number: 81760450; Guangxi Natural Science Foundation of China, Grant/Award Numbers: 2017GXNSFAA198322, 2020JJA140172, 2020JJA140172; The First Batch of High-level Talent Scientific Research Projects of the Affiliated Hospital of Youjiang Medical University for Nationalities in 2019 (Contract No. R20196310). Special Fund of Foshan Summit Plan, grant numbers: 2020B018, 2019D039, 2019D041.

AUTHOR CONTRIBUTIONS

H.L., T.Y., D.H., and X.C. contributed to project administration, study design and data curation. X.L., Q.W., Haineng Huang, Huadong Huang, and H.W. were responsible for investigation and methods. J.W., Q.T., and Q.L. drafted the manuscript. The others performed experiments. All authors have read and approved the final manuscript.

DECLARATION OF INTERESTS

The authors state that there is no conflict of interest in present work.

REFERENCES

1. Malzkorn, B., and Reifenberger, G. (2016). Practical implications of integrated glioma classification according to the World Health Organization classification of tumors of the central nervous system 2016. Curr. Opin. Oncol. *28*, 494–501.
2. Mizumoto, M., Yamamoto, T., Ishikawa, E., Matsuda, M., Takano, S., Ishikawa, H., Okumura, T., Sakurai, H., Matsumura, A., and Tsuboi, K. (2016). Proton beam therapy with concurrent chemotherapy for glioblastoma multiforme: comparison of nimustine hydrochloride and temozolomide. J. Neurooncol. *130*, 165–170.
3. Jhanwar-Uniyal, M., Labagnara, M., Friedman, M., Kwasnicki, A., and Murali, R. (2015). Glioblastoma: molecular pathways, stem cells and therapeutic targets. Cancers (Basel) *7*, 538–555.
4. Hu, Y., Zhu, Q.N., Deng, J.L., Li, Z.X., Wang, G., and Zhu, Y.S. (2018). Emerging role of long non-coding RNAs in cisplatin resistance. OncoTargets Ther. *11*, 3185–3194.
5. Liu, X., Sun, K., Wang, H., and Dai, Y. (2016). Knockdown of retinoblastoma protein may sensitize glioma cells to cisplatin through inhibition of autophagy. Neurosci. Lett. *620*, 137–142.
6. Roberts, N.B., Wadajkar, A.S., Winkles, J.A., Davila, E., Kim, A.J., and Woodworth, G.F. (2016). Repurposing platinum-based chemotherapies for multi-modal treatment of glioblastoma. OncoImmunology *5*, e1208876.
7. Gajski, G., Čimbora-Zovko, T., Rak, S., Osmak, M., and Garaj-Vrhovac, V. (2016). Antitumour action on human glioblastoma A1235 cells through cooperation of bee venom and cisplatin. Cytotechnology *68*, 1197–1205.
8. Galluzzi, L., Senovilla, L., Vitale, I., Michels, J., Martins, I., Kepp, O., Castedo, M., and Kroemer, G. (2012). Molecular mechanisms of cisplatin resistance. Oncogene *31*, 1869–1883.
9. Kulcheski, F.R., Christoff, A.P., and Margis, R. (2016). Circular RNAs are miRNA sponges and can be used as a new class of biomarker. J. Biotechnol. *238*, 42–51.
10. Zhang, S., Liao, K., Miao, Z., Wang, Q., Miao, Y., Guo, Z., Qiu, Y., Chen, B., Ren, L., Wei, Z., et al. (2019). CircFOXO3 promotes glioblastoma progression by acting as a competing endogenous RNA for NFAT5. Neuro-oncol. *21*, 1284–1296.
11. Sun, J., Tian, X., Zhang, J., Huang, Y., Lin, X., Chen, L., and Zhang, S. (2017). Regulation of human glioma cell apoptosis and invasion by miR-152-3p through targeting DNMT1 and regulating NF2 : MiR-152-3p regulate glioma cell apoptosis and invasion. J. Exp. Clin. Cancer Res. *36*, 100.
12. Garofalo, M., and Croce, C.M. (2011). microRNAs: Master regulators as potential therapeutics in cancer. Annu. Rev. Pharmacol. Toxicol. *51*, 25–43.
13. Liu, B., Li, J., Zheng, M., Ge, J., Li, J., and Yu, P. (2017). MiR-542-3p exerts tumor suppressive functions in non-small cell lung cancer cells by upregulating FTSJ2. Life Sci. *188*, 87–95.
14. Zhang, Y., Cong, L., He, J., Wang, Y., Zou, Y., Yang, Z., Hu, Y., Zhang, S., and He, X. (2018). Photothermal treatment with EGFRmAb-AuNPs induces apoptosis in hypopharyngeal carcinoma cells via PI3K/AKT/mTOR and DNA damage response pathways. Acta Biochim. Biophys. Sin. (Shanghai) *50*, 567–578.
15. Zhou, J., Chen, G.B., Tang, Y.C., Sinha, R.A., Wu, Y., Yap, C.S., Wang, G., Hu, J., Xia, X., Tan, P., et al. (2012). Genetic and bioinformatic analyses of the expression and function of PI3K regulatory subunit PIK3R3 in an Asian patient gastric cancer library. BMC Med. Genomics *5*, 34.
16. Huang, W., Ding, X., Ye, H., Wang, J., Shao, J., and Huang, T. (2018). Hypoxia enhances the migration and invasion of human glioblastoma U87 cells through PI3K/Akt/mTOR/HIF-1α pathway. Neuroreport *29*, 1578–1585.
17. Li, X., Wu, C., Chen, N., Gu, H., Yen, A., Cao, L., Wang, E., and Wang, L. (2016). PI3K/Akt/mTOR signaling pathway and targeted therapy for glioblastoma. Oncotarget *7*, 33440–33450.
18. Yu, H.G., Ai, Y.W., Yu, L.L., Zhou, X.D., Liu, J., Li, J.H., Xu, X.M., Liu, S., Chen, J., Liu, F., et al. (2008). Phosphoinositide 3-kinase/Akt pathway plays an important role in chemoresistance of gastric cancer cells against etoposide and doxorubicin induced cell death. Int. J. Cancer *122*, 433–443.

19. Lee, S., Choi, E.J., Jin, C., and Kim, D.H. (2005). Activation of PI3K/Akt pathway by PTEN reduction and PIK3CA mRNA amplification contributes to cisplatin resistance in an ovarian cancer cell line. Gynecol. Oncol. *97*, 26–34.

20. Song, X., Zhang, N., Han, P., Moon, B.S., Lai, R.K., Wang, K., and Lu, W. (2016). Circular RNA profile in gliomas revealed by identification tool UROBORUS. Nucleic Acids Res. *44*, e87.

21. Xu, B., Yang, T., Wang, Z., Zhang, Y., Liu, S., and Shen, M. (2018). CircRNA CDR1as/miR-7 signals promote tumor growth of osteosarcoma with a potential therapeutic and diagnostic value. Cancer Manag. Res. *10*, 4871–4880.

22. Li, Z., and Tzeng, C.M. (2018). Integrated Analysis of miRNA and mRNA Expression Profiles to Identify miRNA Targets. Methods Mol. Biol. *1720*, 141–148.

23. Wang, Y., Kong, X., Guo, Y., Wang, R., and Ma, W. (2017). Continuous dose-intense temozolomide and cisplatin in recurrent glioblastoma patients. Medicine (Baltimore) *96*, e6261.

24. Ahmed, E.M., Bandopadhyay, G., Coyle, B., and Grabowska, A. (2018). A HIF-independent, CD133-mediated mechanism of cisplatin resistance in glioblastoma cells. Cell Oncol. (Dordr.) *41*, 319–328.

25. Ma, B., Yuan, Z., Zhang, L., Lv, P., Yang, T., Gao, J., Pan, N., Wu, Q., Lou, J., Han, C., and Zhang, B. (2017). Long non-coding RNA AC023115.3 suppresses chemoresistance of glioblastoma by reducing autophagy. Biochim. Biophys. Acta Mol. Cell Res. *1864*, 1393–1404.

26. Li, J., Song, J., and Guo, F. (2019). miR-186 reverses cisplatin resistance and inhibits the formation of the glioblastoma-initiating cell phenotype by degrading Yin Yang 1 in glioblastoma. Int. J. Mol. Med. *43*, 517–524.

27. Zhang, X., Wang, S., Lin, G., and Wang, D. (2020). Down-regulation of circ-PTN suppresses cell proliferation, invasion and glycolysis in glioma by regulating miR-432-5p/RAB10 axis. Neurosci. Lett. *735*, 135153.

28. Chen, C., Deng, L., Nie, D.K., Jia, F., Fu, L.S., Wan, Z.Q., and Lan, Q. (2020). Circular RNA Pleiotrophin promotes carcinogenesis in glioma via regulation of microRNA-122/SRY-box transcription factor 6 axis. Eur. J. Cancer Prev. *29*, 165–173.

29. Chen, J., Chen, T., Zhu, Y., Li, Y., Zhang, Y., Wang, Y., Li, X., Xie, X., Wang, J., Huang, M., et al. (2019). circPTN sponges miR-145-5p/miR-330-5p to promote proliferation and stemness in glioma. J. Exp. Clin. Cancer Res. *38*, 398.

30. Yuan, L., Yuan, P., Yuan, H., Wang, Z., Run, Z., Chen, G., Zhao, P., and Xu, B. (2017). miR-542-3p inhibits colorectal cancer cell proliferation, migration and invasion by targeting OTUB1. Am. J. Cancer Res. *7*, 159–172.

31. Li, J., Shao, W., and Feng, H. (2019). MiR-542-3p, a microRNA targeting CDK14, suppresses cell proliferation, invasiveness, and tumorigenesis of epithelial ovarian cancer. Biomed. Pharmacother. *110*, 850–856.

32. Lyu, H., Wang, S., Huang, J., Wang, B., He, Z., and Liu, B. (2018). Survivin-targeting miR-542-3p overcomes HER3 signaling-induced chemoresistance and enhances the antitumor activity of paclitaxel against HER2-overexpressing breast cancer. Cancer Lett. *420*, 97–108.

33. Zhang, W., Liu, Y., Fu, Y., Han, W., Xu, H., Wen, L., Deng, Y., and Liu, K. (2020). Long non-coding RNA LINC00160 functions as a decoy of microRNA-132 to mediate autophagy and drug resistance in hepatocellular carcinoma via inhibition of PIK3R3. Cancer Lett. *478*, 22–33.

34. Nicolau-Neto, P., Da Costa, N.M., de Souza Santos, P.T., Gonzaga, I.M., Ferreira, M.A., Guaraldi, S., Moreira, M.A., Seuánez, H.N., Brewer, L., Bergmann, A., et al. (2018). Esophageal squamous cell carcinoma transcriptome reveals the effect of *FOXM1* on patient outcome through novel PIK3R3 mediated activation of PI3K signaling pathway. Oncotarget *9*, 16634–16647.

35. Luo, J., Yao, J.F., Deng, X.F., Zheng, X.D., Jia, M., Wang, Y.Q., Huang, Y., and Zhu, J.H. (2018). 14, 15-EET induces breast cancer cell EMT and cisplatin resistance by up-regulating integrin αvβ3 and activating FAK/PI3K/AKT signaling. J. Exp. Clin. Cancer Res. *37*, 23.

36. Wang, N., Hou, M.S., Zhan, Y., Shen, X.B., and Xue, H.Y. (2018). MALAT1 promotes cisplatin resistance in cervical cancer by activating the PI3K/AKT pathway. Eur. Rev. Med. Pharmacol. Sci. *22*, 7653–7659.

37. Cai, J., Zhao, J., Zhang, N., Xu, X., Li, R., Yi, Y., Fang, L., Zhang, L., Li, M., Wu, J., and Zhang, H. (2015). MicroRNA-542-3p Suppresses Tumor Cell Invasion via Targeting AKT Pathway in Human Astrocytoma. J. Biol. Chem. *290*, 24678–24688.

38. Huang, X., Li, Z., Zhang, Q., Wang, W., Li, B., Wang, L., Xu, Z., Zeng, A., Zhang, X., Zhang, X., et al. (2019). Circular RNA AKT3 upregulates PIK3R1 to enhance cisplatin resistance in gastric cancer via miR-198 suppression. Mol. Cancer *18*, 71.

39. Xu, Z., Han, X., Ou, D., Liu, T., Li, Z., Jiang, G., Liu, J., and Zhang, J. (2020). Targeting PI3K/AKT/mTOR-mediated autophagy for tumor therapy. Appl. Microbiol. Biotechnol. *104*, 575–587.

40. Lu, R., Zhao, G., Yang, Y., Jiang, Z., Cai, J., and Hu, H. (2019). Inhibition of CD133 Overcomes Cisplatin Resistance Through Inhibiting PI3K/AKT/mTOR Signaling Pathway and Autophagy in CD133-Positive Gastric Cancer Cells. Technol. Cancer Res. Treat. *18*, 1533033819864311.

41. Han, S., Lv, X., Wang, Y., Gong, H., Zhang, C., Tong, A., Zhang, B., and Yao, H. (2015). Effect and mechanism of peroxisome proliferator-activated receptor-γ on the drug resistance of the U-87 MG/CDDP human malignant glioma cell line. Mol. Med. Rep. *12*, 2239–2246.

OMTN, Volume *26*

Supplemental information

circ_PTN contributes to -cisplatin resistance in glioblastoma via PI3K/AKT signaling through the miR-542-3p/PIK3R3 pathway

Hongcheng Luo, Tingzhuang Yi, Deyou Huang, Xiaoping Chen, Xu Li, Qianquan Wan, Haineng Huang, Huadong Huang, Hongyu Wei, Ye Song, Tianshi Que, Rentong Hu, Huatuo Huang, Kunxiang Luo, Chuanyu Li, Chengjian Qin, Chuanhua Zheng, Chuanliu Lan, Wencheng Chen, Dan Zhou, and Qisheng Luo

Supplementary Table 1 Information on the antibodies was presented.

Supplementary Table 1. Information on the antibodies

Antibody	Catalog number	Dilution	Source
Bcl-2	#4223	1/1000	CST, Boston, MA, USA
Caspase-3	#9662	1/1000	CST, Boston, MA, USA
Cleaved Caspase-3	#9661	1/1000	CST, Boston, MA, USA
Caspase-9	#9502	1/1000	CST, Boston, MA, USA
Cleaved Caspase-9	#9505	1/1000	CST, Boston, MA, USA
β-actin	ab8226	1/1000	Abcam, Cambridge, MA, USA
PIK3R3	27035-1-AP	1/500	Proteintech, Chicago, IL, USA
GAPDH	#5174	1/1000	CST, Boston, MA, USA
PI3K-alpha	#4255	1/1000	CST, Boston, MA, USA
p-AKT	ab38449	1/500	Abcam, Cambridge, MA, USA
AKT	ab8805	1/500	Abcam, Cambridge, MA, USA
secondary antibody	ab205718	1/10000	Abcam, Cambridge, MA, USA

Supplementary Table 2 Database accession numbers of new gene/protein sequences were provided.

Supplementary Table 2. Accession Number (Sequences of Genes/Proteins)

**Gene/Protein Name**	**Accession Number of Sequence**
circ_COL1A2	NM_000089
circ_VCAN	NM_004385
circ_PTN	NM_002825
circ_SMO	NM_005631
circ_PLOD2	NM_182943
circ_GLIS3	NM_001042413
circ_EPHB4	NM_004444
circ_CLIP2	NM_003388
Bcl-2	Q92934
Caspase-3	P42574
Cleaved Caspase-3	P42574

Caspase-9	P55211
Cleaved Caspase-3	P55211
β-actin	P60709
CDR1as	NM_004065
cANRIL	NR_003529.3
hsa-miR-223-3p	MIMAT0004570
hsa-miR-432-5p	MIMAT0002814
hsa-miR-620	MIMAT0003289
hsa-miR-1270	MIMAT0005924
hsa-miR-542-3p	MIMAT0003340
AKT1	NM_005163.2
AKT2	NM_001626.6
TSC1	NM_000368.5
IGF1R	NM_000875.5
PIK3R1	NM_181523.3
PIK3R3	NM_003629.4

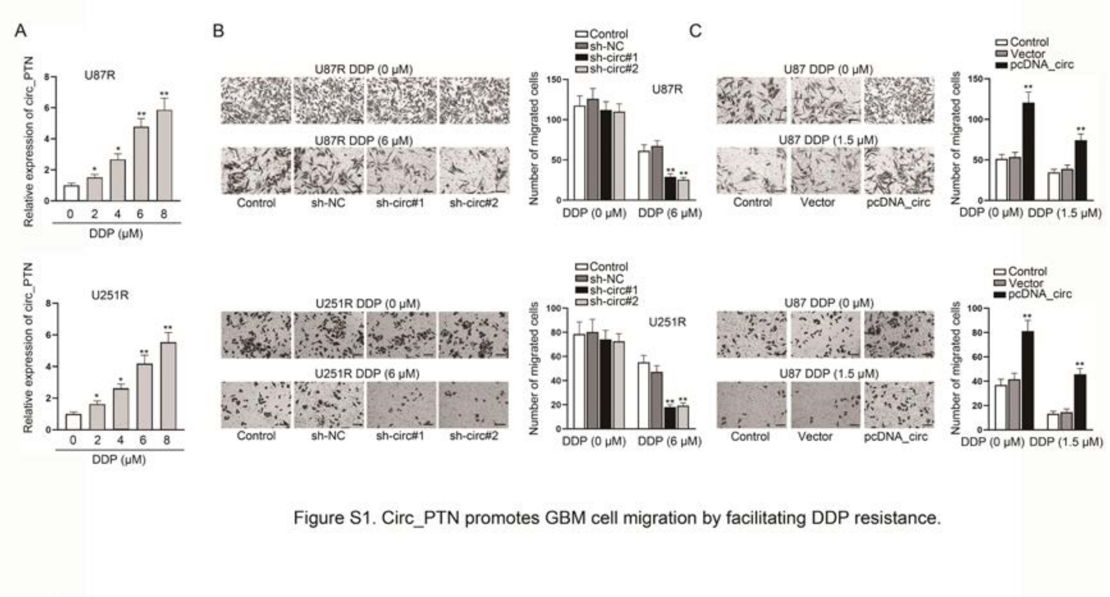

Figure S1. Circ_PTN promotes GBM cell migration by facilitating DDP resistance.

(A). RT-qPCR assay was adopted to detect the RNA level of circ_PTN in DDP-resistant GBM cells treated by different doses of DDP. **(B-C).** Transwell assay

was conducted to test the migration of U87R and U251R cells treated by different doses of DDP upon circ_PTN silencing. *P<0.05, **P<0.01.

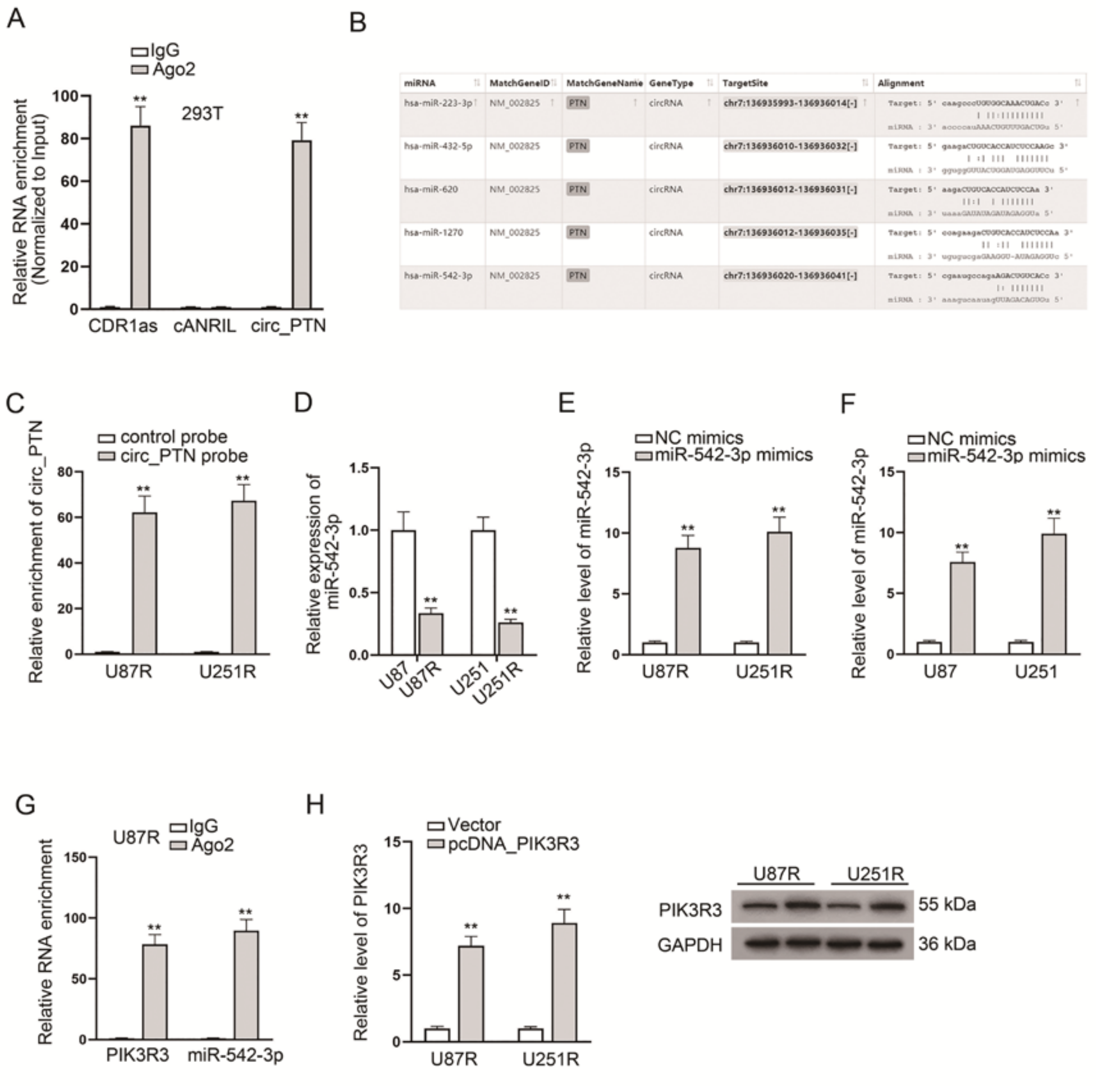

Figure S2. Circ_PTN sponges miR-542-3p to augment PIK3R3 in GBM cells.

Figure S2. Circ_PTN sponges miR-542-3p to augment PIK3R3 in GBM cells.

(A). RIP assay was used to detect the enrichment of CDR1as, cANRIL and circ_PTN in Ago2 groups. **(B).** StarBase was used to predict the miRNAs which could interact with circ_PTN. **(C).** RNA pull down assay was adopted to detect the enrichment of circ_PTN pulled down by circ_PTN probes in DDP-resistant GBM cells. **(D).**

RT-qPCR assay was utilized to analyze the RNA level of miR-542-3p in DDP-resistant GBM cells. **(E-F).** RT-qPCR assay was adopted to examine the RNA level of miR-542-3p in DDP-resistant GBM cells after the transfection with miR-542-3p mimics. **(G).** RIP assay was conducted to analyze the enrichment of PIK3R3 and miR-542-3p in Ago2 groups. **(H).** RT-qPCR and western blot assays were used to examine the mRNA and protein levels of PIK3R3 in DDP-resistant GBM cells. $^{**}P<0.01$.

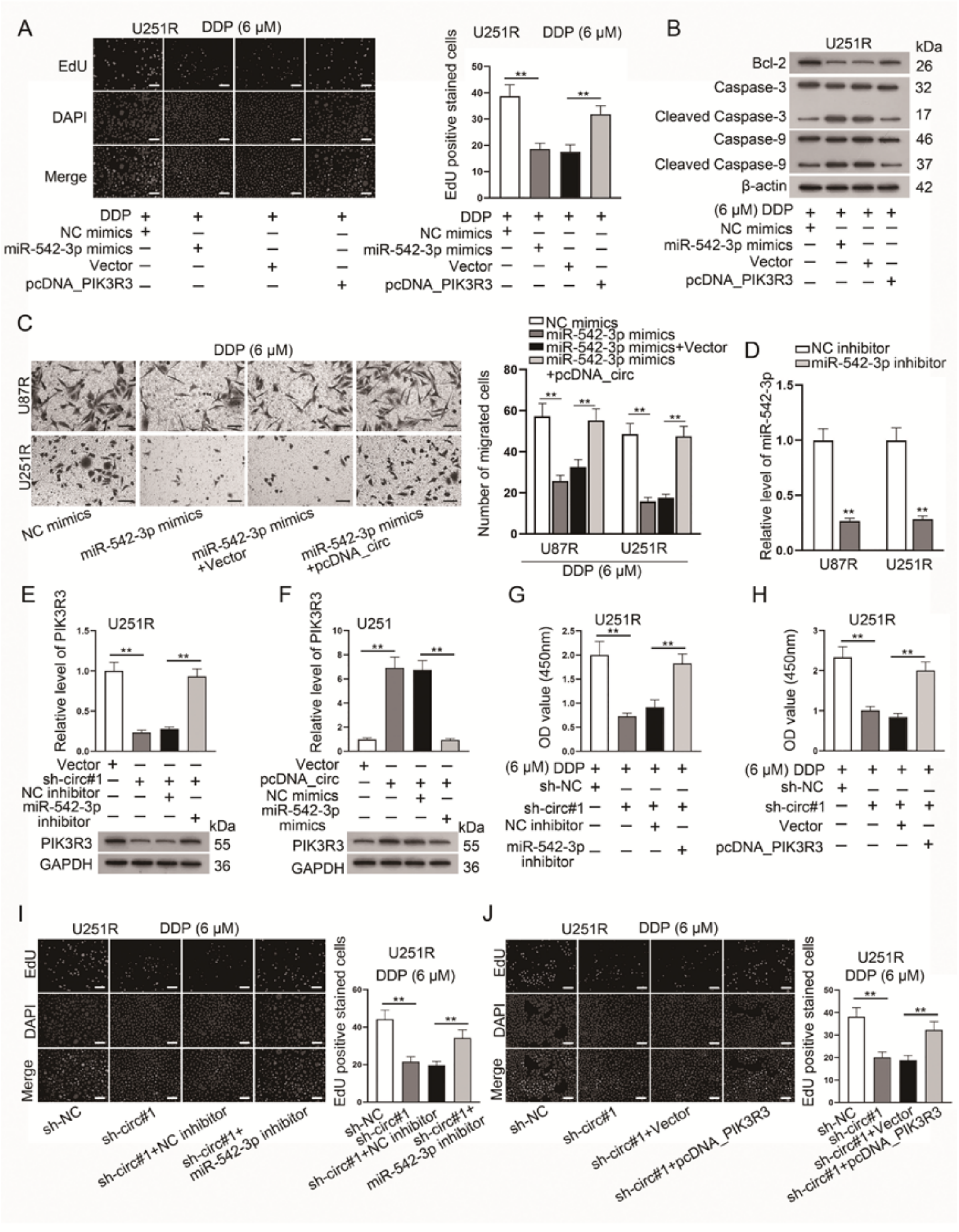

Figure S3. Circ_PTN regulates the DDP resistance via miR-542-3p/PIK3R3 signaling.

Figure S3. Circ_PTN regulates the DDP resistance via miR-542-3p/PIK3R3 signaling.

(A-B). EdU and western blot assays were used to detect proliferation of U251R cells

in different transfection groups and after DDP (6 μM) treatment. **(C).** Transwell assay was utilized to detect the migration of DDP-resistant GBM cells in different transfection groups after the treatment of 6 μM DDP. **(D).** RT-qPCR assay was used to test the RNA level of miR-542-3p in DDP-resistant GBM cells in different transfection groups. **(E-F).** RT-qPCR and western blot assays were used to detect the mRNA and protein levels of PIK3R3 in U251R and U251 cells in different transfection groups. **(G-H).** CCK-8 assays were adopted to examine the viability of DDP-resistant U251R cells in different transfection groups after the treatment of 6 μM DDP. **(I-J).** EdU assays were utilized to detect the proliferation of U251R cells in different transfection groups after the treatment of 6 μM DDP. **P<0.01.

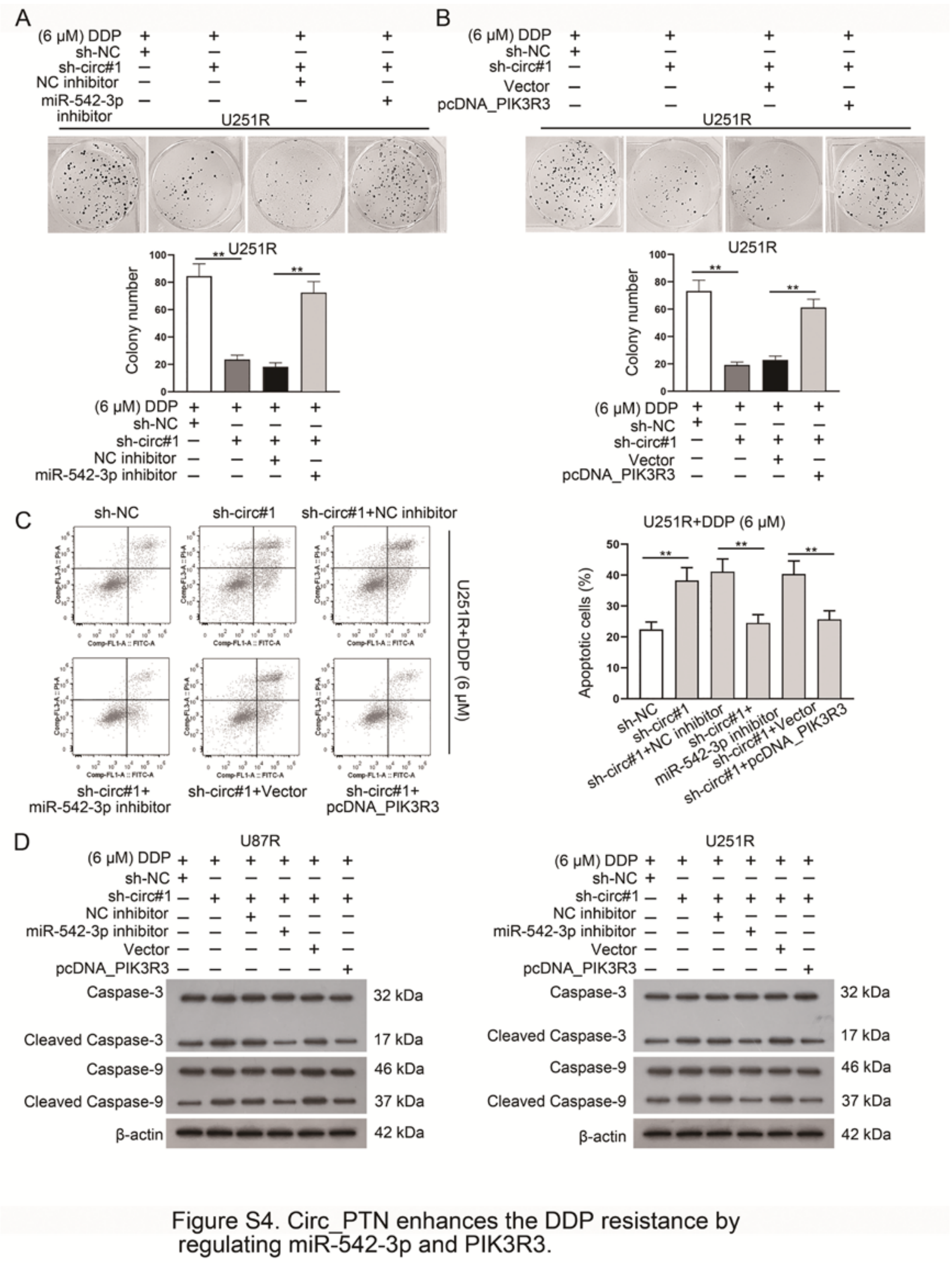

Figure S4. Circ_PTN enhances the DDP resistance by regulating miR-542-3p and PIK3R3.

(A-B). Colony formation assays were conducted to examine the proliferation of U251R cells in different transfection groups after the treatment of 6 μM DDP. **(C-D).**

Flow cytometry and western blot assays were carried out to examine the apoptosis of DDP-treated U251R cells under different transfection conditions. **P<0.01.

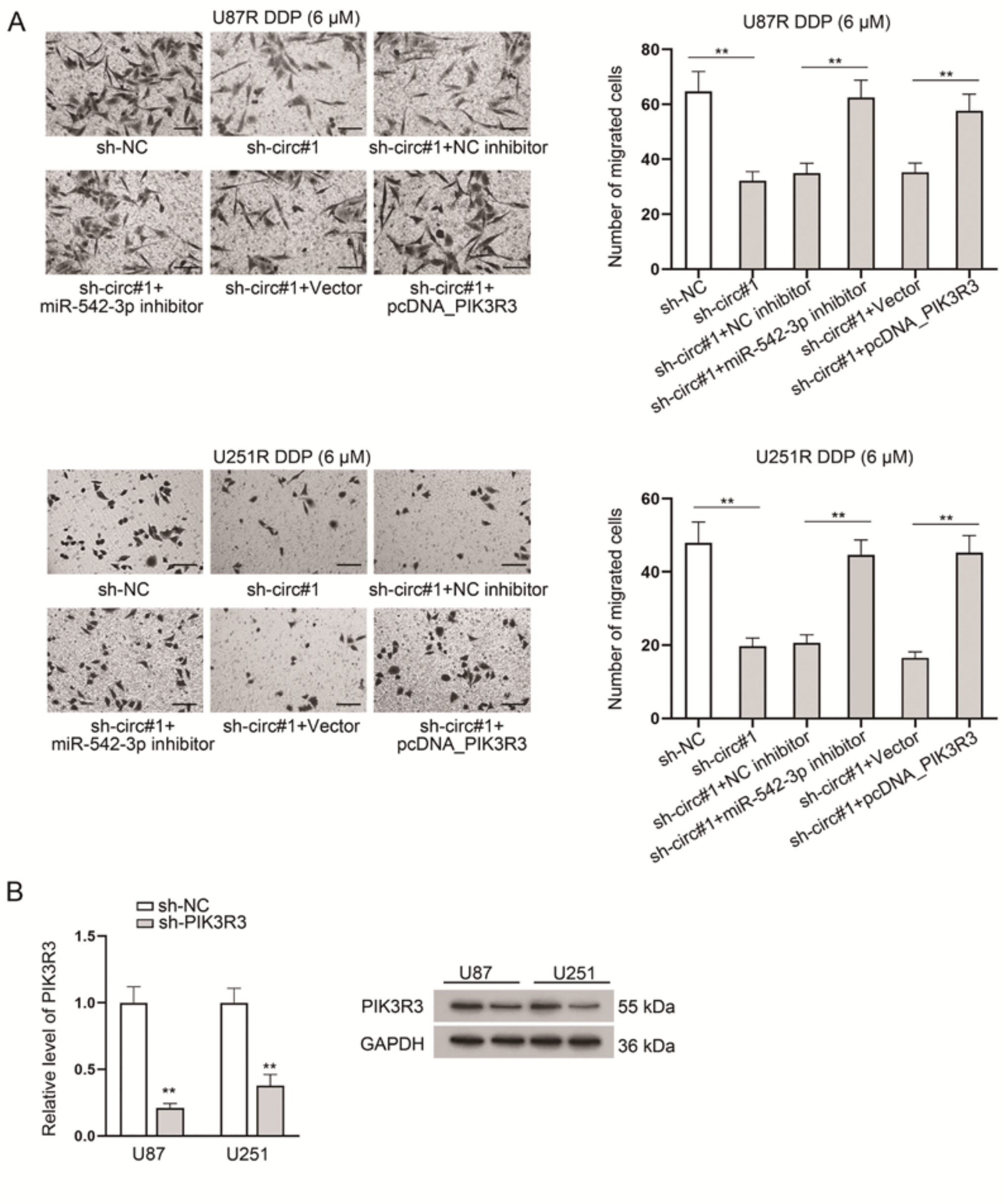

Figure S5. Circ_PTN/miR-542-3p/PIK3R3 axis contributes to GBM cell migration under DDP treatment.

Figure S5. Circ_PTN/miR-542-3p/PIK3R3 axis contributes to GBM cell migration under DDP treatment.

(A). Transwell assays were used to analyze the migration of DDP-resistant GBM cells in different transfection groups after the treatment of 6 μM DDP. **(B).** RT-qPCR and

western blot assays were utilized to test the mRNA and protein levels of PIK3R3 in U87 and U251 cells transfected with sh-NC or sh-PIK3R3. **P<0.01.
